# Elastic Properties and Enhanced Piezoelectric Response at Morphotropic Phase Boundaries

**DOI:** 10.3390/ma8125452

**Published:** 2015-12-02

**Authors:** Francesco Cordero

**Affiliations:** CNR-ISC, Istituto dei Sistemi Complessi, Area della Ricerca di Roma-Tor Vergata, Via del Fosso del Cavaliere 100, Roma I-00133, Italy; francesco.cordero@isc.cnr.it; Tel.: +39-06-4993-4114; Fax: +39-06-4993-4076

**Keywords:** piezoelectricity, Morphotropic Phase Boundary, elasticity, polarization rotational instability, PZT, Pb-free ferroelectrics, anelasticity, domain walls

## Abstract

The search for improved piezoelectric materials is based on the morphotropic phase boundaries (MPB) between ferroelectric phases with different crystal symmetry and available directions for the spontaneous polarization. Such regions of the composition x−T phase diagrams provide the conditions for minimal anisotropy with respect to the direction of the polarization, so that the polarization can easily rotate maintaining a substantial magnitude, while the near verticality of the $T_{\mathrm{MPB}}\left(x\right)$ boundary extends the temperature range of the resulting enhanced piezoelectricity. Another consequence of the quasi-isotropy of the free energy is a reduction of the domain walls energies, with consequent formation of domain structures down to nanoscale. Disentangling the extrinsic and intrinsic contributions to the piezoelectricity in such conditions requires a high level of sophistication from the techniques and analyses for studying the structural, ferroelectric and dielectric properties. The elastic characterization is extremely useful in clarifying the phenomenology and mechanisms related to ferroelectric MPBs. The relationship between dielectric, elastic and piezoelectric responses is introduced in terms of relaxation of defects with electric dipole and elastic quadrupole, and extended to the response near phase transitions in the framework of the Landau theory. An account is provided of the anelastic experiments, from torsional pendulum to Brillouin scattering, that provided new important information on ferroelectric MPBs, including PZT, PMN-PT, NBT-BT, BCTZ, and KNN-based systems.

## 1. Introduction

The ferroelectric (FE) material most used in applications is PbZr${}_{1-x}$Ti${}_{x}$O${}_{3}$ (PZT). It started to be studied in the early 1950s, when in its x−T phase diagram a nearly vertical boundary between ferroelectric (FE) rhombohedral (R) and tetragonal (T) phases was found [1] and called morphotropic phase boundary (MPB). Soon it was found that the piezoelectric coefficients $d_{33}$ and $d_{31}$ versus composition at room temperature are sharply peaked at the MPB composition x≃0.47 [2]. Since then the study and search of MPBs also in other perovskite solid solutions (of the type A${}_{1-x}^{\prime }$A${}_{x}^{\prime \prime }$B${}_{1-y}^{\prime }$B${}_{y}^{\prime \prime }$O${}_{3}$ or more complicated) have never ceased to be pursued, with a burst of activities in the last 25 years.

A MPB between ferroelectric phases with different available directions for the spontaneous polarization is favorable to a high piezoelectric response for two reasons: because the transverse susceptibilities near the phase transition are enhanced and because have better thermal stability. In fact, if a phase transition occurs between a tetragonal (T) phase with polarization $P_{T}$ along $\left(001\right)$ and an often rhombohedral (R) phase with $P_{R}$ along $\left(111\right)$, an instability sets in, that is transversal with respect to the original direction of $P_{T}$. This is illustrated by the fact that the free energy, in addition to the minima for $P||\left[100\right]$ in the T phase, must develop minima for $P||\left[111\right]$, requiring a flattening along the directions joining the two types of minima, perpendicularly to $\left[100\right]$. This is a transverse or rotational instability of the polarization, and is accompanied by an enhancement of the transverse dielectric susceptibility, shear piezoelectric constant and shear elastic compliance. The enhanced piezoelectric response can be exploited in applications, but when the transition is driven by a change of temperature, namely by the crossing of a thermotropic phase boundary (TPB), the enhancement of the susceptibilities is peaked only near the transition temperature. A well known example is BaTiO${}_{3}$ with its sequence during cooling of cubic paraelectric (C-PE), T-, orthorhombic- (O) and R-FE phases. A comparison of BaTiO${}_{3}$ with PbTiO${}_{3}$, the latter lacking the rotational FE transitions, can be found in [3], in terms of the concepts useful in the present review.

A better thermal stability of the transversally unstable state with enhanced piezoelectric response is found if the FE/FE transition is driven by a change of composition *x*, rather than temperature. In the ideal case, the MPB between T-FE and R-FE phases is vertical in the x−T phase diagram. In practice, a truly vertical MPB may be difficult to find, but the materials with the best piezoelectric properties are solid solutions of perovskites with T-FE and R-FE or O-FE phases, separated by a boundary with strong inclination, a good approximation of a MPB with transverse instability of the polarization.

The above explanation sounds simple, but the subject has been studied with so many experimental techniques and from varying theoretical points of view that it is actually much more complex and no synthesis is yet found among the various approaches. For a long time the common opinion was that the coexistence of R and T phases at the MPB allowed an easy reorientation of the polarization among the possible 14 $\langle \pm 1,0,0\rangle$ and $\langle \pm 1,\pm 1,\pm 1\rangle$ pseudocubic directions [4,5]. This was already disproven by the observation that in PZT the maximum electromechanical response occurs in the tetragonal phase just outside the two-phase MPB region [6], but the discovery by Noheda *et al.* [7] that PZT in a narrow region to the Zr-rich side of the MPB is monoclinic (M) rather than R gave a great impulse to the research on the origin of the enhanced piezoelectric response near MPBs. In addition, the requirement from EU environmental regulations of substituting toxic Pb in PZT, still now the most used piezoelectric material, triggered a search of Pb-free solid solutions with MPB between T-FE and R-FE phases, in order to reproduce or improve the properties of PZT. These are the main motives behind the extensive and intensive studies on the relationship between MPB and high piezoelectric response.

The traditional electric and electromechanical characterizations of FE materials have been complemented with many structural investigations with increasing levels of sophistication, including synchrotron X-ray diffraction, neutron diffraction, TEM, spectroscopies like Raman and anelastic spectroscopies. Each technique provides information on a specific spatial and frequency range and level of correlation of the atomic motions, and the picture that emerges is quite complicated.

Most of the rich literature on the MPB and its effect on piezoelectricity can be divided in essentially two categories, to which we will refer as the “monoclinic phase and polarization rotation” and the “adaptive phase”. The “M phase” school began with the discovery by Noheda and coworkers of the M phase at the MPB of PZT [7], soon followed by similar findings at the MPBs of other PT based perovskites with exceptionally high piezoelectric activities [8]. This discovery fitted perfectly with the almost simultaneous theoretical analysis of the high piezoelectricity of PZN-PT in terms of rotation of the polarization rather than domain wall motion [9], since the M phase of PZT corresponds to the intermediate, often called “bridging”, state between R and T phases, if the polarization rotates under the application of a strong electric field. These findings and interpretation elicited considerable experimental and theoretical activities aimed at perfecting the model and searching materials with improved properties, but soon after also a completely different explanation of the same facts was proposed [10]. According to the “adaptive phase” school, the M phase is actually nanotwinned R and/or T, often called “miniaturized domains”, where the direction of the average polarization can be continuously changed by modulating the separations between the twin boundaries and therefore the fractions of the T or R variants with different direction of the spontaneous polarization. Such a phenomenon is well known for ferroelastic martensitic alloys, and the proliferation of twin boundaries would be possible thanks to the vanishing anisotropy energy at the MPB. On the scale of most experiments, including X-ray and neutron diffraction, the adaptive phase appears as homogeneous with lower symmetry intermediate between the variants of which is composed. Since then many observations of domain miniaturization at MPB of PT-based and Pb-free ferroelectric perovskites have been reported and the debate on the validity of one or the other paradigms is still in progress.

In synthesis, at the MPB between FE-R and FE-T phases the minima of the free energy for P along the $\langle 100\rangle$ and $\langle 111\rangle$ directions are degenerate, implying in a first approximation a completely isotropic free energy, or at least rather flat with respect to changes of the direction of the spontaneous polarization (see Section 4.3). According to the “M phase” school this flattening of the free energy allows an easy rotation of P and the stabilization of the associated low symmetry M phase. On the other hand, according to the “adaptive phase” school, the low barrier for the rotation of P implies low energy for the walls between domains that remain R or T, and it is the exceptionally high density and mobility of such walls the responsible for the high piezoelectricity.

The two views are apparently quite different from each other, but when analyzing the various experiments in order to find a conclusive evidence for one or the other, it appears that the differences are blurred. Indeed, both a M phase and the proliferation of twin walls are a result of the isotropization of the free energy near the MPB, and it would be no surprise that they coexist. The issue becomes to evaluate the relative contributions of the intrinsic enhancement to the susceptibilities from an intermediate phase and the extrinsic contribution from the motion of the domain walls (DW).

Several review articles and book chapters have already appeared on the subject of the high piezoelectricity at MPBs [11,12,13,14,15,16,17,18,19,20]. In the present review the focus is on the elastic properties. That a large elastic compliance favors a large piezoelectric strain is well known, and a roughly inverse relationship is found plotting for many materials the electromechanical coupling versus the Young’s modulus or, perhaps more significantly, the ratio of the shear to longitudinal moduli [21]*.* The present review is an attempt at explaining the contribution that the study of the elastic and anelastic properties can offer to the comprehension of the mechanisms and complex phenomenology associated with FE MPBs.

It is first shown that the piezoelectric response contains the contributions of the dielectric susceptibility and elastic compliance with equal weight. The expected contributions to these susceptibilities is very cursorily discussed for the motion of the domain walls and more in depth for the intrinsic response at phase transitions, within the framework of the Landau theory. Then, a survey is offered of the experimental picture of the phases involved in the MPB region of phase diagrams of PZT, other Pb-based and finally Pb-free perovskites, with emphasis on the elastic properties.

Much of the physics and phenomenology usually associated with the topic of piezoelectricity at a MPB does not depend on the fact that the boundary between the two phases with different spontaneous polarization is a really vertical MPB in the temperature versus composition phase diagram. Already in the prototype PZT, the MPB can be crossed by varying temperature and its near verticality has only the role of increasing the thermal stability of the state with enhanced piezoelectricity. This is especially true in Pb-free materials, where the boundaries involving a rotation of the spontaneous polarization are generally normal TPB [22]. Also, these cases will be included in the present review, which instead does not cover MPBs between FE and AFE phases.

## 2. Dielectric, Elastic and Piezoelectric Responses

The piezoelectric response is closely related to both the dielectric susceptibility *χ* and elastic compliance *s*. In fact, while the dielectric susceptibility gives the electric polarization P as response to an applied electric field E,

$$P_{i}=\chi_{ij}E_{j}\;,$$

and the elastic compliance gives the strain *e* due to a stress *σ*,

$$e_{ij}=s_{ijkl}\sigma_{kl}\;,$$

the piezoelectric coefficient is the mixed response of strain due to the application of an electric field,

$$e_{ij}=d_{ijk}E_{k}$$

(converse piezoelectric effect) and polarization or displacement field $D=P+\varepsilon_{0}E$ to a stress, $D_{i}=d_{ijk}\sigma_{jk}$ (direct piezoelectric effect) [23,24,25]. Since the ferroelectric materials treated here have $\chi \gg \varepsilon_{0},$ the permittivity of vacuum, the dielectric susceptibility *χ* and permittivity $\varepsilon =\varepsilon_{0}+\chi ≃\chi$ of the material will not be distinguished. In order to clarify the relationship between piezoelectric coefficients and the dielectric and elastic susceptibilities, and set the basis for the discussion of the intrinsic versus extrinsic contributions to the piezoelectric effect, the responses are first derived for the case of paraelastic and paraelectric relaxation of independent point defects having both electric dipole and elastic quadrupole. The resulting expressions are valid both for the intrinsic instantaneous response, obtained in the limit of null relaxation time, and for relaxing defects. Next we will generalize to the case of extended defects like domain walls (DW).

### 2.1. Paraelectric, Paraelastic and Mixed Piezoelectric Relaxation: Thermodynamics

The classical treatment of anelastic, dielectric and piezoelectric relaxation from a uniform molar concentration *c* of point defects can be found in the seminal works of Nowick and Heller [26,27] and more recently in Damjanovic [28]. For simplicity, let us consider point defects with crystallographically equivalent orientations *α*, having electric dipole $\mu^{\left(\alpha \right)}$ with components $\mu_{i}^{\left(\alpha \right)}$ and elastic quadrupole (or dipole, see below) $\lambda^{\left(\alpha \right)}$ with components $\lambda_{ij}^{\left(\alpha \right)}$. For example, in a perovskite ABO${}_{3}$ a pair of an acceptor B${}^{\prime }$ in B with an O vacancy (V${}_{\text{O}}$) can have the V${}_{\text{O}}$ along the six possible cubic directions α=±x,±y,±z, which are equivalent in the cubic paraelectric phase. The treatment can be extended to include non-equivalent states, also considered as reactions among different defects [29], and defect clusters [30].

The electric dipole $\mu_{i}^{\left(\alpha \right)}$ will have in general more possible orientations of the elastic quadrupole $\lambda_{ij}^{\left(\alpha \right)}$, because the application of inversion changes the sign of the electric dipole of the defect but leaves unchanged its local distortion, which, being a 2nd rank tensor, is centrosymmetric [23,24]. In the above example of the tetragonal B${}^{\prime }-$V${}_{\text{O}}$ pair in the cubic perovskite, applying inversion to $\mu^{\left(+z\right)}=\left(0,0,\mu \right)$ transforms it into $\mu^{\left(-z\right)}=\left(0,0,-\mu \right),$ but both pairs have the same

$$\lambda^{\left(\pm z\right)}=\lambda^{\left(z\right)}=\left(\begin{array}{ccc} \lambda_{2} & 0 & 0\\ 0 & \lambda_{2} & 0\\ 0 & 0 & \lambda_{1} \end{array}\right)\;.$$

Therefore, the tetragonal defect in a cubic crystal has six possible orientations for the electric dipole, which can be represented as an arrow pointing in the α=±x,±y,±z directions, but only three for the elastic quadrupole, which can be represented as an ellipsoid with the major (or minor) axis along α=x,y,z. The latter is usually called “elastic dipole”, for analogy with the dielectric case [26,27], and because it is the first moment of multipolar expansion of the deformation due to the defect (see [31], where it can be derived from the double force tensor $P_{ij}$ as $\lambda_{ij}=s_{ijkl}P_{kl}$) but has the symmetry of an electric quadrupole and it is also called “elastic quadrupole” [32,33].

As a final simplification, let us consider a one-dimensional case of defects with only two possible states α=1,2 (like B${}^{\prime }-$V${}_{\text{O}}$ pairs with only *x* and *z* orientations and fields applied along one of the two directions). We want to find expressions for the contributions of the defects to the susceptibilities Δs=de/dσ, Δχ=dP/dE and Δd=de/dE. On application of an electric field *E* and stress *σ* the electric and elastic (mechanical) energies of the defects become

$$\begin{array}{ccc} W_{e}^{\left(\alpha \right)} & = & -\mu^{\left(\alpha \right)}E\\ W_{m}^{\left(\alpha \right)} & = & -v_{0}\lambda^{\left(\alpha \right)}\sigma \\ W^{\left(\alpha \right)} & = & W_{e}^{\left(\alpha \right)}+W_{m}^{\left(\alpha \right)} \end{array}$$

where $v_{0}$ is the cell (molecular) volume. If $\mu^{\left(2\right)}-\mu^{\left(1\right)}=\Delta \mu$, $\lambda^{\left(2\right)}-\lambda^{\left(1\right)}=\Delta \lambda$, ΔW= $W^{\left(2\right)}-W^{\left(1\right)},$ then the equilibrium values of the populations $n_{\alpha }$, with $n_{1}+n_{2}=1$ and $n_{2}-n_{1}=\Delta n$, follow the Boltzmann distribution, ${\bar{n}}_{\alpha }\propto \exp\left(-W^{\left(\alpha \right)}/k_{B}T\right)$ so that

(1) $$\bar{\Delta n}=\tanh\left(-\Delta W/2k_{B}T\right)$$

The change in population is reflected in the total strain

$$e=c\left(n_{1}\lambda^{\left(1\right)}+n_{2}\lambda^{\left(2\right)}\right)=c\left[\frac{\left(n_{1}+n_{2}\right)}{2}\left(\lambda^{\left(1\right)}+\lambda^{\left(2\right)}\right)+\frac{\left(n_{2}-n_{1}\right)}{2}\left(\lambda^{\left(2\right)}-\lambda^{\left(1\right)}\right)\right]$$

split into constant and variable parts, of which only the last one is interesting for the dynamical susceptibility

(2) $$de=c\frac{\Delta n}{2}\Delta \lambda$$

and similarly for the polarization per unit volume

$$dP=\frac{c}{v_{0}}\frac{\Delta n}{2}\Delta \mu$$

After application of a static dσ, the equilibrium elastic response is

(3) $$\Delta s=\frac{de}{d\sigma }=c\frac{1}{2}\frac{d\Delta n}{dW}\frac{dW}{d\sigma }\Delta \lambda =\frac{cv_{0}{\left(\Delta \lambda \right)}^{2}}{2k_{\text{B}}T\cosh^{2}\left(\Delta W/2k_{\text{B}}T\right)}$$

where the $\cosh^{2}\left(\Delta W/2k_{\text{B}}T\right)≃1$ for $\Delta W\ll 2k_{\text{B}}T$ is put in evidence, in case the inequality does not hold. In fact, when dealing with relaxation of point defects among equivalent orientations, like B${}^{\prime }-$V${}_{\text{O}}$ pairs in the cubic phase of PbTiO${}_{3}$, the inequality holds, and the anelastic relaxation strength depends on temperature as 1/T. Instead, if the relaxation is among inequivalent states, like B${}^{\prime }-$V${}_{\text{O}}$ pairs in the FE-T phase of PbTiO${}_{3}$, ΔW may be comparable or even much larger than $k_{B}T$, and the relaxation strength is accordingly abated. In fact, in these conditions the application of stress causes little redistribution of the populations, since the state of higher energy remains very little populated. Having made these remarks, we simply write the elastic response for the usual case $\Delta W\ll 2k_{\text{B}}T$ as

$$\Delta s=\frac{de}{d\sigma }=\frac{cv_{0}{\left(\Delta \lambda \right)}^{2}}{2k_{\text{B}}T}.$$

Following the same steps, we can write the electric response as

$$\Delta \chi =\frac{dP}{dE}=\frac{c}{v_{0}}\frac{d\Delta n}{2dW}\frac{dW}{dE}\Delta \mu =\frac{c{\left(\Delta \mu \right)}^{2}}{2v_{0}k_{\text{B}}T}.$$

and the mixed piezoelectric response as

$$\Delta d=\frac{de}{dE}=c\frac{d\Delta n}{2dW}\frac{dW}{dE}\Delta \lambda =\frac{c\Delta \lambda \Delta \mu }{2k_{\text{B}}T}=\frac{dP}{d\sigma }\;.$$

From these expressions it is clear that both the electric and elastic responses contribute with equal weight to the piezoelectric one, which is their geometrical mean:

(4) $$\Delta d=\sqrt{\Delta s\Delta \chi }$$

The above relationships have been derived for molar concentrations *c* of defects after reaching thermal equilibrium through jumping with a characteristic time *τ* for a time much longer than *τ*. The same formulas can be made valid also for the intrinsic response of the ferroelectric cells, by setting c=1, and changing the 1/T dependence into Curie-Weiss $1/\left(T-T_{C}\right)$, in order to take into account the correlation among the cells in the mean field approximation (see [34]; a derivation along these lines for *χ* of BaTiO${}_{3}$ can be found in [35], while for the purely elastic case in [36]). It should be taken into account that the relationship Equation (4) regards only the contributions to the three susceptibilities from the spontaneous polarization and does not hold for the total susceptibilities *d*, *χ* and *s*. In fact, in a FE phase d≃Δd, χ≃Δχ because $d_{0}$ and $\chi_{0}$ of the paraelectric phase are null or negligible, but $s=s_{0}+\Delta s$ and the background lattice compliance $s_{0}$ of the PE phase is not negligible compared to Δs.

### 2.2. Paraelectric and Paraelastic Relaxation: Kinetics

In order to derive the dynamic susceptibilities, let us consider fields with angular frequency *ω*, $E=E_{0}e^{-i\omega t}$, $\sigma =\sigma_{0}e^{-i\omega t}$; the complete response, for example elastic, from the intrinsic susceptibility $s_{0}$ and the retarded paraelastic one from Δs given by Equation (1) is

$$e=\left(s_{0}+\Delta s\right)\sigma$$

Also the populations and all the variables vary sinusoidally with the same frequency, but with a phase lag due to the retarded response of the defects. This is deduced from the rate equations for the instantaneous populations

(5) $$\left\{\begin{array}{c} {\dot{n}}_{1}=-\nu_{21}n_{1}+\nu_{12}n_{2}\\ {\dot{n}}_{2}=+\nu_{21}n_{1}-\nu_{12}n_{2} \end{array}\right.\;$$

with the condition $n_{1}+n_{2}=$ 1 and the rates usually but not necessarily following the Arrhenius law. After a little manipulation, the above rate equations can be written in terms of Δn= $n_{2}-n_{1}$ as

(6) $$\frac{d\Delta n}{dt}=-\left(\nu_{12}+\nu_{21}\right)\left[\Delta n-\left(\frac{\nu_{21}-\nu_{12}}{\nu_{12}+\nu_{21}}\right)\right]$$

and the second term in square brackets can be rewritten in terms of the equilibrium populations ${\bar{n}}_{i}$. In fact, from Equation (5) $0=-\nu_{21}{\bar{n}}_{1}+\nu_{12}{\bar{n}}_{2}=-\nu_{21}{\bar{n}}_{1}+\nu_{12}\left(1-{\bar{n}}_{1}\right)$, that can be solved for ${\bar{n}}_{1}=\frac{\nu_{12}}{\nu_{12}+\nu_{21}}$ and similarly ${\bar{n}}_{2}=\frac{\nu_{21}}{\nu_{12}+\nu_{21}}$. Inserting in the above equation we find

(7) $$\begin{array}{ccc} \frac{d\Delta n}{dt} & = & -\frac{\Delta n-\bar{\Delta n}}{\tau } \end{array}$$

$$\begin{array}{ccc} \tau^{-1} & = & \nu_{12}+\nu_{21} \end{array}$$

Of course, it is possible to verify that the equilibrium populations deduced from the thermodynamic and kinetic argument coincide. It is convenient to express the rate equation in terms of the deviation $\Delta n^{\prime }$ from $\bar{\Delta n}\left(\sigma =0\right)$:

(8) $$\begin{array}{ccc} \Delta n^{\prime } & = & \Delta n-\bar{\Delta n}\left(\sigma =0\right) \end{array}$$

(9) $$\begin{array}{ccc} {\bar{\Delta n}}^{\prime } & = & \bar{\Delta n}-\bar{\Delta n}\left(\sigma =0\right)=\frac{d\bar{\Delta n}}{dW}\frac{dW}{d\sigma }\sigma ≃\frac{v_{0}\Delta \lambda }{2k_{\text{B}}T}\sigma \end{array}$$

In this manner, Equation (7) becomes $d\Delta n^{\prime }/dt=-\tau^{-1}\left(\Delta n^{\prime }-{\bar{\Delta n}}^{\prime }\right)$ or $\left(1+i\omega \tau \right)\Delta n^{\prime }={\bar{\Delta n}}^{\prime }$, so that

$$\Delta n^{\prime }=\frac{{\bar{\Delta n}}^{\prime }}{1+i\omega \tau }=\frac{v_{0}\Delta \lambda }{2k_{\text{B}}T}\sigma$$

can be inserted in the expression of total strain (instantaneous elastic $s_{0}\sigma$ and anelastic de of Equation (2))

(10) $$e=s_{0}\sigma +\Delta \lambda \frac{\Delta n^{\prime }}{2}=s_{0}\sigma +\frac{v_{0}{\left(\Delta \lambda \right)}^{2}}{4k_{\text{B}}T}\frac{1}{1+i\omega \tau }\sigma$$

and finally the dynamic compliance e/σ can be written as

(11) $$s=s^{\prime }-is^{\prime \prime }=s_{0}+\frac{v_{0}{\left(\Delta \lambda \right)}^{2}}{4k_{\text{B}}T}\frac{1}{1+i\omega \tau }$$

In the same manner

(12) $$\chi =\chi^{\prime }-i\chi^{\prime \prime }=\chi_{0}+\frac{{\left(\Delta \mu \right)}^{2}}{4k_{\text{B}}T}\frac{1}{1+i\omega \tau }$$

and

(13) $$d=d^{\prime }-id^{\prime \prime }=d_{0}+\frac{v_{0}\Delta \lambda \Delta \mu }{4k_{\text{B}}T}\frac{1}{1+i\omega \tau }$$

These expressions are Debye relaxations whose relaxation time *τ* is the reciprocal mean transition frequency between the possible states (Equation (7)). The frequency dispersion

(14) $$f\left(\omega \tau \right)=\frac{1}{1+i\omega \tau }=\frac{1}{1+{\left(\omega \tau \right)}^{2}}-i\frac{\omega \tau }{1+{\left(\omega \tau \right)}^{2}}$$

is a step centered at ωτ=1 for the real part and a peak centred at ωτ=1 for the imaginary part, or the semicircle of radius 1 in the Cole-Cole plot $-f^{\prime \prime }$ *vs.* $f^{\prime }$. Relaxations of these type are characteristic of diluted non-interacting point defects.

### 2.3. Non-Debye Relaxation From Domain Walls

The Debye relaxation of the previous paragraph can be generalized to extended defects by introducing a spectrum of relaxation times and properly choosing the relaxation strengths. In the case of the motion of domain walls, the spectrum of relaxation times derives from the distribution of the lengths of the DW free to move between pinning points, that can be intersections with other walls, impurities and other defects or grain boundaries. Regarding the relaxation strengths, *μ* represents the change of polarization due to the fact that the area swept by the DW moving from domain *α* into domain *β* changes from $P^{\left(\alpha \right)}$ to $P^{\left(\beta \right)}$, and *λ* represents the change of strain from $e^{\left(\alpha \right)}$ to $e^{\left(\beta \right)}$. Similarly to the point defect case, the motion of a $180^{{}^{\circ}}$ wall produces dielectric but not anelastic relaxation, because domains with $P^{\left(\alpha \right)}=$ $-P^{\left(\beta \right)}$ have $e^{\left(\alpha \right)}=$ $e^{\left(\beta \right)}$. Therefore, only the motion of non-$180^{{}^{\circ}}$ DW produces anelastic and piezoelectric relaxation or response.

**Figure 1:**
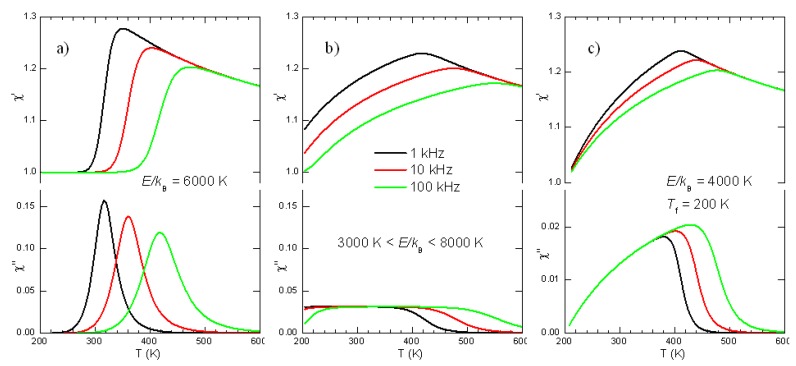
Real part and imaginary parts of the susceptibilities following Equation (15), with the three types of distributions of relaxation time: (**a**) single time with activation energy $E/k_{B}=6000$ K; (**b**) uniform distribution between 3000 K and 8000 K; (**c**) uniform distribution between $\tau_{0}$ and Vogel-Fulcher law.

In most of the experiments considered here, the susceptibilities are measured as a function of temperature at various frequencies. The Debye relaxations Equations (11)–(13) produce peaks in the mechanical, dielectric and piezoelectric losses centred at the temperatures $T_{\max}$ for which the condition $\omega \tau \left(T_{\max}\right)≃1$ is satisfied and steps in the real parts at the same temperatures. Since *τ* generally follows the Arrhenius law τ= $\tau_{0}\exp\left(W/k_{\text{B}}T\right)$, these spectra measured *vs. T* shift to higher *T* when measured at higher *ω* (Figure 1a). In the case of DW, the broadening from the distribution of relaxation times may be so important to change considerably the spectrum. Figure 1 shows two such examples based on uniform distributions of $g\left(\ln\tau \right)$, that can be easily solved and written in closed form:

(15) $$\chi =\chi^{\prime }-i\chi^{\prime \prime }=\chi_{0}+\frac{\Delta }{k_{\text{B}}T}\int_{0}^{\infty }d\left(\ln\tau \right)\frac{g\left(\ln\tau \right)}{1+i\omega \tau }$$

where the uniform distribution $g\left(\ln\tau \right)=1/\ln\left(\tau_{2}/\tau_{1}\right)$ is normalized between $\tau_{1}$ and $\tau_{2}$ and null outside; then

$$\begin{array}{ccc} \chi^{\prime \prime } & = & \frac{\Delta }{k_{\text{B}}T\ln\left(\tau_{2}/\tau_{1}\right)}\left[\arctan\omega \tau_{2}-\arctan\omega \tau_{1}\right]\\ \chi^{\prime } & = & \chi_{0}+\frac{\Delta }{k_{\text{B}}T}\left[1-\frac{1}{2\ln\left(\tau_{2}/\tau_{1}\right)}\ln\left(\frac{{\left(\omega \tau_{2}\right)}^{2}+1}{{\left(\omega \tau_{1}\right)}^{2}+1}\right)\right]\;. \end{array}$$

In Figure 1 the three examples are obtained with the following choices: (a) $\tau_{1}=\tau_{2}=\tau_{0}\exp\left(E/k_{\text{B}}T\right)$ with $\tau_{0}=10^{-12}$ s and $E/k_{\text{B}}=$ 6000 K, *i.e.*, the single time Debye relaxation; (b) $\tau_{1}=\tau_{0}\exp\left(3000\;\text{K}/T\right)$, $\tau_{2}=\tau_{0}\exp\left(8000\;\text{K}/T\right)$ (c) corresponding to a broad distribution of activation energies; $\tau_{1}=\tau_{0}$, $\tau_{2}=\tau_{0}\exp\left[E/k_{\text{B}}\left(T-T_{f}\right)\right]$ with $T_{f}=200$ K, corresponding to glassy dynamics with freezing at $T_{f}$.

Relaxations from DW motion do not generally conform to any of these approximations for various reasons. For example the relaxation strength ∝1/T assumes relaxation between states with the same energy, except for the influence of the applied stress, and assumes a temperature independent density of DW (for DW properties see e.g., [37,38]); nevertheless, some remarks are possible. The broadening of the spectrum certainly hinders its shift in temperature with varying frequency, especially in the imaginary part, but does not suppress it. Unless the distribution of activation energies is extremely broad, the frequency dispersion in the real part remains well recognizable below the temperature satisfying $\omega \tau \left(T\right)∼1$ for the slowest *τ*. Therefore, a characteristic of the extrinsic contribution of DW to the susceptibilities, including the piezoelectric response, is some frequency dispersion in the real part, at least at the low temperatures where the DW motion starts freezing.

## 3. Methods for Measuring the Elastic Properties

A recent review of methods for measuring the elastic properties of materials, not including the free resonance and piezoresonance methods, can be found in [39].

Methods that measure resonance frequencies *f* or sound velocities *v* actually measure a combination of elastic constants *c* and density *ρ*, since both *f* and *v* are $\propto \sqrt{c/\rho }$. Yet, when presenting the curves as a function of temperature, *ρ* is usually considered as constant with respect to the much larger variations of *c*. Therefore, the temperature dependencies of the elastic moduli *c* or compliances *s* will be presented normalized to a reference value, generally in the PE phase, as

$$\frac{c}{c_{0}}=\frac{s_{0}}{s}=\left(\frac{f^{2}}{f_{0}^{2}}\right)\;\text{or}\;\left(\frac{v^{2}}{v_{0}^{2}}\right)$$

**Figure 2:**
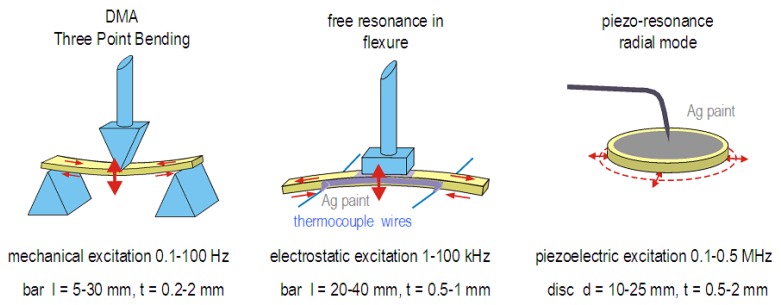
Three common geometries for measuring the dynamic Young’s modulus along the sample length or radially. Also indicated are typical sizes of the samples and driving/resonance frequencies.

Regarding the contributions of the various elastic constants to the effective moduli, let us consider the Young’s modulus *Y* (generally indicated as E, but here as *Y* in order to avoid confusion with the electric field) and shear modulus *G*. The first is the elongation of a bar pulled along its length, and can be measured also in flexure, which consists in nonuniform compression in the concave side and extension in the convex side (Figure 2). If $\hat{n}$ is the crystallographic direction along the bar length, then for a cubic crystal [40]

(16) $$\begin{array}{ccc} Y{\left(\hat{n}\right)}^{-1} & = & s_{11}-2\Gamma \left(s_{11}-s_{12}-\frac{1}{2}s_{44}\right) \end{array}$$

(17) $$\begin{array}{ccc} \Gamma & = & n_{1}^{2}n_{2}^{2}+n_{2}^{2}n_{3}^{2}+n_{1}^{2}n_{3}^{2} \end{array}$$

The shear modulus *G* can be measured from the torsion angle of a bar along whose length is applied a torque; if $\hat{n}$ is the crystallographic direction along the bar length, then

(18) $$G{\left(\hat{n}\right)}^{-1}=s_{44}+4\Gamma \left(s_{11}-s_{12}-\frac{1}{2}s_{44}\right)$$

When measuring a ceramic, under the assumption of uniform stress from grain to grain, one can set the orientational average $\bar{\Gamma }=\frac{1}{5}$ into the previous expressions and find the Reuss polycrystalline averages

(19) $$\begin{array}{ccc} \bar{Y^{-1}} & = & \frac{3}{5}s_{11}+\frac{2}{5}s_{12}+\frac{1}{5}s_{44} \end{array}$$

(20) $$\begin{array}{ccc} \bar{G^{-1}} & = & \frac{4}{5}\left(s_{11}-s_{12}\right)+\frac{3}{5}s_{44} \end{array}$$

Even though *G* has no contribution from the bulk modulus, both *Y* and *G* contain contributions from all the elastic constants.

The presence of internal degrees of freedom, e.g., domain walls and defects moving with their characteristic rate, introduces a retarded elastic response and hence an imaginary component in the dynamic modulus and compliance and dissipation of the elastic energy. For a sample in free or forced vibration the elastic energy loss coefficient is given by [40]

$$Q^{-1}=\tan\phi =\frac{s^{\prime \prime }}{s^{\prime }}=\frac{c^{\prime \prime }}{c^{\prime }}$$

where *Q* is the mechanical quality factor and *ϕ* the phase lag between excitation and response.

### 3.1. Piezoelectric Resonance

The method is described in detail elsewhere in this Special Issue, and only some essential information is provided here for completeness. The method consists in exciting the mechanical resonances through the piezoelectric effect of the same sample, applying the ac electric field with an impedance bridge. With appropriate combinations of sample geometries it is possible to measure the full set of linear electric, mechanical, and electromechanical coefficients, and all related losses of a piezoelectric ceramic material [41,42].

Piezoresonance for measuring the elastic constants may be of complicated quantitative interpretation, because it is affected by the level of P alignment, which decreases on approaching $T_{C}$ (see e.g., [43]). In principle it should vanish in the paraelectric (PE) phase, but in practice some residual or precursor polarization may exist also above $T_{C}$, making it possible to extend the measurements into the PE phase.

### 3.2. Free Flexural Resonance

The sample is a long (l= 20−50 mm) and thin (h= 0.2−1.5 mm) bar suspended on thin thermocouple wires in correspondence with the nodes for the 1st flexural resonance [44,45]. It is made conductive with Ag paint and electrostatically excited by an electrode close to its surface (Figure 2). The method is particularly suited to measure the low amplitude limit behavior of the compliance, in the linear and amplitude independent range. This is possible thanks to the high sensitivity of the method for revealing the sample vibration through the modulation of the sample-electrode capacitance. It is generally possible during the same run to excite the first three odd flexural modes, whose resonant frequencies are in the ratios $f_{1}:f_{3}:f_{5}=1:5.4:$13.2. The frequency range is 100 Hz −100 kHz. The real part of the Young’s modulus is deduced from

$$Y^{-1}=s=\frac{1}{\rho }{\left(\frac{1.028}{f_{1}}\frac{h}{l^{2}}\right)}^{2}$$

while the elastic energy loss coefficient

$$Q^{-1}=Y^{\prime \prime }/Y^{\prime }=s^{\prime \prime }/s^{\prime }\;$$

is deduced from the width of the resonance peaks in forced oscillations or from the decay of the free oscillations. Very low losses ($<10^{-6}$) may be measured, thanks to the weak clamping by wires.

### 3.3. DMA

The Dynamic Mechanical Analyzer is now being adopted by an increasing number of laboratories. Under many respects it is the most effective method, since with instruments available on the market it is possible to measure the elastic dynamic modulus controlling temperature, frequency in a broad range of ≤0.1 Hz to ∼100 Hz and in principle the vibration amplitude. In addition, various geometries are possible, but the most common for rigid samples is the three point bending shown in Figure 2. In practice sensitivity and noise issues require displacements of at least few microns, but too large displacements break the samples, so that the available range of strain is restricted to around $10^{-4}$ (see e.g., [46]). This is a large displacement, likely causing nonlinear effects in the presence of domain boundaries. In addition, the contact of the sample with the probe and the holder and the subtraction of their contribution from the measured strain may introduce instrumental effects that are difficult to separate from the sample contribution. For these reasons, a comparison between anelastic spectra obtained with DMA and free resonance may require the consideration of nonlinear effects and parasitic losses. Yet, the large amplitudes with the DMA are closer to those involved in some applications, and from this point of view the spectra obtained with the DMA may be more informative. In addition, it is possible to use an insulating (e.g., quartz) holder and probe and through thin wires attached to the upper and lower electroded sample faces it is possible to measure the flexoelectric effect [47].

### 3.4. Torsion Pendulum

Another method in a similar frequency range is the inverted torsion pendulum, where the sample is a bar clamped at the extremities and vertically placed in the furnace/cryostat at the end of a long rigid rod, with weights in the upper room temperature region for regulating the torsional inertia and hence resonance frequency, and pulled with a wire and counterweight in order to minimize and control the tension on the sample. The torsional force is applied with electromagnets and the angle is measured with the optical lever. This method invented by Kê [48] and, together with the free flexural resonance adopted by Bordoni [44], was the first method for anelastic measurements.

### 3.5. Resonant Ultrasound Spectroscopy

A recent review on the results obtained by Resonant Ultrasound Spectroscopy (RUS) in perovskites, including the ferroelectric and multiferroic ones, can be found in [49]. The sample is a parallelepiped also of very small size, clamped between two piezoelectric transducers (through buffer rods for maintaining the transducers to room temperature), one for excitation and one for detection, at two opposite corners. At each temperature the whole resonance spectrum is recorded and analyzed in order to extract some combinations of elastic constants from the resonance frequencies and the losses from the widths of the resonances. The frequency range is 0.1–2 MHz.

### 3.6. Ultrasound Propagation

The pulse-echo method, described in many books (e.g., [50]) consists in attaching a piezoelectric transducer with grease or glue to the sample, sending ultrasound pulses, with typical frequencies of 1–100 MHz, and receiving back the reflected impulse. From the travel time one measures the sound velocity, while from the amplitude decay the absorption. The method is particularly effective on single crystals, in order to get the whole set of elastic constants, and covers an important frequency range for studying fluctuations in phase transitions [51].

### 3.7. Brillouin Scattering

To the upper end of the frequency range, 10 GHz and above, is Brillouin scattering, where a light beam passing through the sample is diffracted by phonons, and one observes photons with energies higher or lower than the incident beam, depending whether a phonon has been created or absorbed. The quality of the measurements has improved so much to be comparable with the other methods, in measuring both the sound velocity and absorption, without the problems of clamping and large applied stress and at much higher frequencies [52,53].

The shift $\nu_{B}$ of the scattered light with respect to the incident beam is proportional to the velocity *v* of the sound wave with which it has interacted, $\nu_{B}\propto v$, the latter is proportional to some combination of the elastic constants, e.g., the longitudinal acoustic (LA) mode along $\left(100\right)$ has $v_{\mathrm{LA}}\left(100\right)=$ $\sqrt{c_{11}/\rho }.$ The full-width at half maximum (FWHM) of the line of scattered light is instead proportional to the acoustic absorption of that mode.

## 4. Landau Theory of Phase Transitions

### 4.1. First and Second Order Ferroelectric Transitions

The standard approach for studying the ferroelectric phase transitions, the related anomalies in the susceptibilities and the phase diagrams is the theory of Landau, as described in many articles and books [54,55]. Here is given a brief account, in order to provide a self-contained exposition of the concepts used in the rest of the article.

Near a phase transition the free energy is expanded in a series of powers of the order parameter (OP), the physical quantity that defines the phase transformation and is null in the high temperature symmetric phase. For ferroelectrics the OP is the polarization P, initially considered as a one-dimensional order parameter, e.g., $P=P_{3}$ of a T-FE phase

(21) $$\begin{array}{ccc} G_{P} & = & \frac{\alpha }{2}P^{2}+\frac{\beta }{4}P^{4}+\frac{\gamma }{6}P^{6}+..\; \end{array}$$

(22) $$\begin{array}{ccc} \alpha & = & \alpha^{\prime }\left(T-T_{C}\right) \end{array}$$

**Figure 3:**
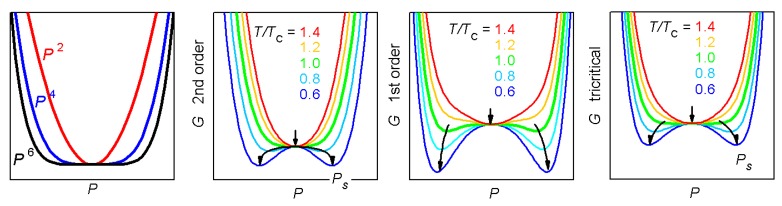
Evolution of $G\left(P,T\right)$ at $T/T_{C}=0.6$, 0.8, 1, 1.2, 1.4 for three choices of the expansion coefficients in Equation (21): $\alpha^{\prime }=1$ and β=0.7, γ=0 (2nd order); β=−0.6, γ=0.7 (1st order); β=−0.1, γ=0.7 (near to tricritical).

Odd powers of *P* are forbidden by symmetry in the C-PE phase, because it is invariant with respect to inversion, and inversion would change the sign of such terms. The standard approach, certainly valid close to the temperature $≃T_{C}$ of the transition, is to consider all the coefficients constant except for *α*, which is assumed to decrease linearly with *T* and become negative below $T_{C}.$ In this manner, the free energy $G\left(P\right)$ has an absolute minimum at P=0, representing the PE phase, but below $T_{C}$ the quadratic parabola becomes negative and other minima develop at $\pm P_{s}$, the spontaneous polarization. The parabolas $P^{2n}$ become flatter and flatter with increasing the power and the evolution of $G_{P}$ with decreasing *T* are shown in Figure 3. The magnitude of $P_{s}$ can be found by solving the equilibrium condition in the absence of external field, here shown for the case of null *γ* and higher order coefficients

$$\begin{array}{ccc} 0 & = & {\left.\frac{\partial G}{\partial P}\right|}_{P_{s}}=\left(\alpha +\beta P_{s}^{2}\right)P_{s}\\ P_{s}^{2} & = & \left\{\begin{array}{c} \;0\;\;\\ \alpha^{\prime }\left(T_{C}-T\right)/\beta \end{array}\right.\begin{array}{c} \;\left(T>T_{C}\right)\;\;\\ \left(T<T_{C}\right) \end{array} \end{array}$$

The susceptibility can be deduced from $\chi^{-1}=\frac{\partial E}{\partial P}$ and $E=\frac{\partial G}{\partial P}$, so that

(23) $$\chi^{-1}=\frac{\partial^{2}G}{\partial P^{2}}\overset{\text{2nd}\;\text{order}}{=}\alpha +3\beta P_{s}^{2}=\left\{\begin{array}{c} \alpha^{\prime }\left(T_{C}-T\right)\;\;\\ -2\alpha^{\prime }\left(T_{C}-T\right) \end{array}\right.\begin{array}{c} \;\left(T>T_{C}\right)\;\;\\ \left(T<T_{C}\right) \end{array}$$

where it is reminded that the final expression is valid for a 2nd order transition with expansion truncated to the 4th power of *P*. It is reassuring that the choice of the expansion (Equation (21)) yields exactly the Curie-Weiss law for *χ*.

It is also possible to reproduce 1st order transitions by setting β<0 and including at least γ>0. In that case there is a temperature region where the three minima of $G_{P}$ at P=0 and $P=\pm P_{s}$ coexist, corresponding to the coexistence of PE and FE phases. A comparison of the evolution of the free energy in the two cases is shown in Figure 3, in order to point out that for the 2nd order transition, where the minimum at P=0 gradually splits in the two minima, the bottom of the free energy is more flat than in the 1st order case, where there is always some deep minimum. This means that the susceptibility χ, which is the reciprocal of the curvature of *G* (Equation (23)), diverges at $T_{C}$ at a 2nd order transition, but it does not for a 1st order one, unless this is close to 2nd order. The latter case occurs when β→0 and is called tricritical; it is associated with a particularly flat free energy (see the green curves at $T_{C}$ in Figure 3) that causes a marked divergence of the susceptibility. Notice that the divergence as $|T-T_{C}{|}^{-1}$ implies that the enhancement of the susceptibility fades rapidly away from $T_{C}$, and the same holds for the piezoelectric coefficient.

### 4.2. Coupling to Stress: The Electrostrictive and Piezoelectric Effects

When dealing with elastic compliance and piezoelectric effect, we have to introduce the stress $\sigma_{ij}$ and strain $e_{ij}$ variables in the free energy expansion and, in order to simplify the notation, we adopt the single index Voigt notation: 11→1, 22→2, 33→3, 23→4, 13→5, 12→6. The elastic compliance *s* and stiffness *c* are

(24) $$\begin{array}{ccc} s_{ij} & = & \frac{de_{i}}{d\sigma_{j}} \end{array}$$

(25) $$\begin{array}{ccc} c_{ij} & = & \frac{d\sigma_{i}}{de_{j}} \end{array}$$

and the elastic energy is $dW_{el}=\sigma_{i}de_{i}$, where the convention of summing over repeated indexes is understood and maintained in the rest of the article. The elastic energy $\frac{1}{2}c_{ij}e_{i}e_{j}$ has to be added to $G_{P},$ but we choose to use $\sigma_{i}$ instead of $e_{i}$ as independent variables and therefore use the Gibbs instead of the Helmholtz free energy, G=F−σe, so that

(26) $$G_{el}=-\frac{1}{2}s_{ij}^{0}\sigma_{i}\sigma_{j}$$

where $s_{ij}^{0}$ is the compliance in the PE phase (or with clamped *P*).

Since terms odd in *P* are forbidden in the PE phase, the piezoelectric coefficients that couple linearly *σ* and *P* cannot be introduced in the Landau expansion, and the next admissible terms are

(27) $$G_{c}=-Q_{ijk}\sigma_{i}P_{j}P_{k}-R_{ijkl}\sigma_{i}\sigma_{j}P_{k}P_{l}-\ldots$$

where $Q_{ijk}$ are the electrostrictive coefficients in Voigt notation for the stress. In order to derive piezoelectric coefficients of the type

$$d_{ik}=de_{i}/dE_{k}\;,$$

consider the effect of the electric field E on the spontaneous strain $e_{s}$ in the FE phase. Strain is derived from *G* as

$$e_{i}=-\frac{\partial G}{\partial \sigma_{i}}=s_{ij}^{0}\sigma_{j}+Q_{ijk}P_{j}P_{k}$$

and $e_{s}$ is obtained by setting $P=P_{s}$ and σ=0

(28) $$e_{s,i}=Q_{ijk}P_{s,j}P_{s,k}\;.$$

so that

(29) $$d_{ik}=\frac{de_{s,i}}{dE_{k}}=Q_{ijl}\left(\chi_{jk}P_{s,l}+P_{s,j}\chi_{lk}\right)\;.$$

According to this expression, piezoelectricity appears in the FE phase as electrostriction that is biased by the spontaneous polarization.

### 4.3. The Polarization Anisotropy

In order to reproduce FE phases with various symmetries starting from the C-PE phase, it is necessary to include in the free energy expansion (Equation (21)) terms anisotropic in P. Since odd powers of any component $P_{i}$ are not allowed, the lowest order such term is

(30) $$G_{\mathrm{an}}=\beta_{\mathrm{an}}\left(P_{1}^{2}P_{2}^{2}+P_{2}^{2}P_{3}^{2}+P_{1}^{2}P_{3}^{2}\right)$$

**Figure 4:**
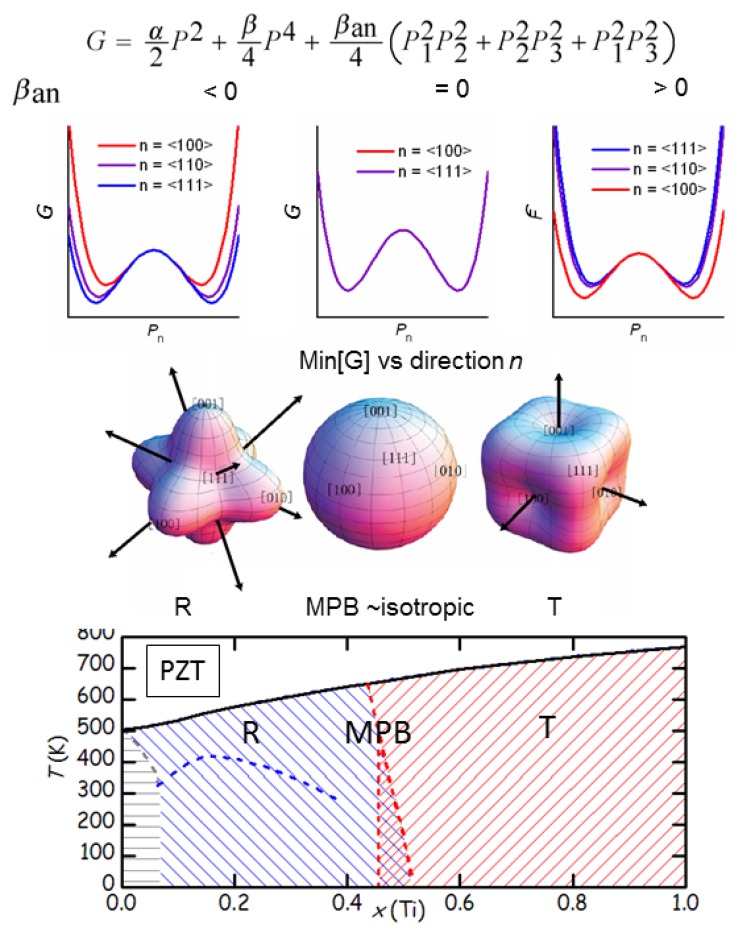
Effect of the 4th order anisotropic term on the minima of the free energy (adapted from [56]). When the Ti fraction in PbZr${}_{1-x}$Ti${}_{x}$O${}_{3}$ (PZT) increases through the morphotropic phase boundaries (MPB) at x≃0.5, the R phase obtained with $\beta_{\mathrm{an}}<0$ changes into T obtained with $\beta_{\mathrm{an}}>0$.

Figure 4 shows how the minima of $G\left(P\right)$ depend on $\beta_{\mathrm{an}}$ for $P||\langle 100\rangle ,$ $\langle 110\rangle$ and $\langle 111\rangle$ and the angular plots of $\min\left(G\right)$. When $\beta_{\mathrm{an}}>$ 0 the absolute minima are along $\langle 100\rangle$ and reproduce the T phase, while for $\beta_{\mathrm{an}}<$ 0 the absolute minima are along $\langle 111\rangle$ reproducing the R phase; the intermediate situation $\beta_{\mathrm{an}}=0$ is isotropic. The minima for $P||\langle 110\rangle$, corresponding to a O phase, are never absolute; they can become absolute if 6th order terms are included in $G_{\mathrm{an}}$ [57]. Thermodynamically stable M phases must be described by absolute minima along lower symmetry directions, that can be obtain by including anisotropic coefficients of 8th and higher order [58,59,60].

We can now describe the situation near the MPB, e.g., of PZT, as follows: when the Ti fraction increases through the MPB at x≃0.5, the R phase requires $\beta_{\mathrm{an}}<0$ and changes into T with $\beta_{\mathrm{an}}>0$; the intermediate situation at the MPB is the isotropic one, meaning that there is no change of energy when the polarization changes direction, In other words, $G\left(P\right)$ still has deep minima when varying the magnitude *P*, so that the longitudinal susceptibilities are not affected, but becomes flat for changes ⊥P, the so-called rotational or transverse instability with divergence of the transverse and shear susceptibilities.

According to Khachaturyan, Rossetti and the school of the adaptive phase, the 4th order anisotropic term Equation (30) describes all the physics of ferroelectrics like PZT at their MPB. The near-isotropy at the MPB implies a negligible DW energy between the various R and T domains (of course, some residual anisotropy must exist in order to ensure that domains have P along $\langle 100\rangle$ and $\langle 111\rangle$ and not anywhere). A negligible DW energy allows for a proliferation of DW with the formation of a so-called adaptive phase, where the domains are so small that on the scale of X-ray, neutron diffraction and most other experiments it appears monoclinic.

### 4.4. Phase Diagrams From Landau Free Energies

The Landau expansion of the free energy is in principle valid only in the symmetric phase and close to the transition it describes, but it is actually used also far from it, in order to calculate the whole phase diagram in the temperature, composition and pressure spaces. When successive phase transitions are to be reproduced, as is the case of the C-PE→T−FE followed by T−FE→R−FE, terms of higher order have to be included in the expansion, in order to reproduce the various minima corresponding to the different FE phases. Doubts exist on the validity of modulating the free energy surface with many high order terms, if other sources of local variation of the free energy exist, such as compositional fluctuations and compatibility strains of the different domains [61]. Especially in the case of low symmetry M phases requiring anisotropic terms of 8th and higher order, such an approach is considered invalid by the school of the adaptive phase, on the basis that the apparent low symmetry arises from nanoscale twinning, also called domain miniaturization [56,62], as found in martensitic alloys [63].

On the other hand, it should also be considered that intrinsic anisotropies, not necessarily in high symmetry directions, may be induced by the competition of long and short range atomic interactions from directional bonds, especially when atoms with lone pair like Pb and Bi are present [64,65]. The case of the high pressure M phase of PbTiO${}_{3}$ [66,67], which certainly lacks compositional fluctuations, provides supports to the existence of an intrinsically M phase and hence to the validity of the free energy expansions up to 8th and higher order [58].

In order to reproduce x−T phase diagrams from a free energy expansion, its coefficients must be dependent on the composition according to some criterion. In principle they should also depend on temperature, as discussed in [68], and concerns on the validity of the standard assumption of constant coefficients except for *α* in Equation (21) have been expressed also in relation to FE perovskites [69,70]. As a loose indication on the range of validity of the Landau expansion we may consider the conformity of the dielectric susceptibility to the Curie-Weiss law, which is obtain under the usual assumption on the expansion coefficients (Equation (23)). Yet, the analysis of the nonlinear dielectric response of the classical BaTiO${}_{3}$ has shown that an eighth-power anharmonic polarization term is necessary and it must also depend on temperature [70].

An early and extensive effort to reproduce the x−T phase diagram of PZT and its main physical properties like dielectric susceptibility, polarization, strain, *etc.* was carried out by Haun and coworkers in a series of articles [71,72]. At that time the M phase at the MPB had not yet been discovered, but otherwise the whole phase diagram was reproduced, including the O-AFE phase at Zr-rich compositions and the low temperature tilting of the octahedra. The free energy expansion therefore included FE and AFE polarization and the antiferrodistortive rotations of the octahedra, up to the 6th order.

More theoretically oriented approaches have also been adopted for studying the R/T MPB region of the phase diagram of PZT, with fewer parameters and features than in the works of Haun.

There is an early thermodynamic analysis of Isupov [73], based on the Landau expansion up to the 6th powers of $P_{i}$. He considered FE T, R and O phases and recognized that the MPB is the locus of the points where the 4th order anisotropy vanishes, a concept amply developed later.

**Figure 5:**
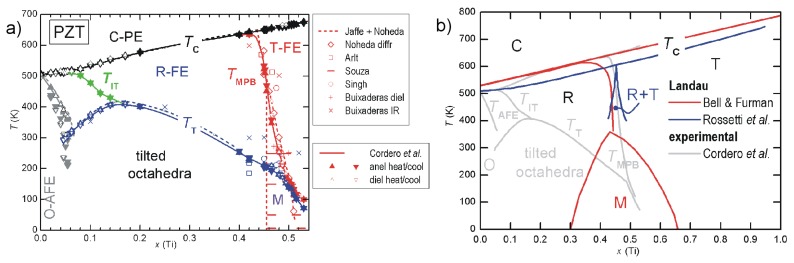
(**a**) phase diagram of PZT deduced from several experimental techniques. Thick lines and triangles: anelastic and dielectric, Cordero *et al.* [76,77,78,79,80]; dashed lines: standard phase diagram of Jaffe *et al.* [4] with revision of Noheda [8]; additional MPB points from dielectric, Arlt [81]; Raman, Souza *et al.* [82]; XRD, Singh *et al.* [83]; IR reflectivity, Buixaderas *et al.* [84] (**b**) two examples of phase diagrams obtained with the Landau theory truncated to the 6th order and (i) using a double order parameter in order to obtain an intermediate M phase (Bell & Furman [74]); (ii) only coexistence of R and T phase (Rossetti *et al.* [85]); the grey lines are from the experimental phase diagram on the left.

In order to reproduce the main features of the MPB of PZT, Bell and Furman [74] ignore the O-AFE phase of PbZrO${}_{3}$ and octahedral tilting, and consider a combination of two OPs p and q up to the 6th power, that, together with two sets of coefficients extracted from the work of Haun, reproduce the two ends of the phase diagram: PbTiO${}_{3}$ for x=1 and a fictitious FE PbZrO${}_{3}$ extrapolated from R-FE PZT for x=0. At intermediate compositions *x* they linearly interpolate between the two sets of coefficients and $T_{C}\left(x\right)$, with the additional introduction of coupling coefficients of the allowed mixed p and q terms. The flexibility from the possible coexistence of the two sets of polarizations allows all the M phases to be obtained also if the expansion is limited to the 6th power. With the coefficients appropriate for PZT, only the monoclinic M${}_{A}$ phase (see Section 7.3), the one actually observed, is reproduced and the amplitude of the region it occupies depends on the choice of the coupling terms. An example of phase diagram they obtained is shown in Figure 5, where an interesting feature of their model appears: the $T_{\mathrm{RT}}\left(x\right)$ line always bends sharply toward x=0 before approaching $T_{C}$. This feature is generally not found or ignored in the experimental works, except [75,76], as we shall see later. It is also numerically found that the major contribution to the enhanced piezoelectric coefficient near the MPB is due to the mixed pq terms associated with the M phase, rather than p and q separately.

A different approach to obtain M phases as stable solutions of the minimization of the free energy is to include anisotropic coefficients of 8th and higher order in the free energy [58,59]. The justification for the important role played by such high order terms is proposed to be quenched chemical disorder [58], though a M phase has subsequently found in chemically homogeneous PbTiO${}_{3}$ under pressure [67].

Also Rossetti *et al.* [85] discussed the topology of the phase diagram by linearly interpolating the parameters at the PbZrO${}_{3}$ and PbTiO${}_{3}$ end points, but, at variance with Bell and Furman [74], following the idea that the M phase is actually an adaptive phase of R and T nanodomains. Accordingly, in the free energy expansion in terms of P, 6th and higher order anisotropic terms were neglected, and the remaining 4th order anisotropy changes sign when passing from the T-FE to the R-FE side. As a consequence, two tricritical points are found along the $T_{C}\left(x\right)$ line, where the FE/PE transition passes from 2nd order near the MPB to 1st order far away from it. An example of phase diagram obtained in this manner and adapted to PZT is also shown in Figure 5. Notice the absence of the deflection of the MPB line on approaching $T_{C}$. A recent thermodynamic analysis of several experimental phase diagrams presenting a MPB between R and T phases is presented in [86], where the M phase is obtained by including terms up to the 8th order in P.

The issue of the tricritical points along the $T_{C}\left(x\right)$ border with isotropic free energy had already been discussed by Haun [87] and is further considered in other papers in connection with the enhancement of the piezoelectric activity. Porta and Lookman [88], expanded the work of Rossetti *et al.* [85] by including the 6th powers of P and, beside analyzing the effect of the various coefficients on the position of the tricritical points, studied the deviation of the MPB from a vertical line.

## 5. Susceptibilities Near a Phase Transition: Landau Theory

### 5.1. Elastic Anomaly at a 2nd Order Phase Transition From Landau Theory

A general expression for the anomalies caused by phase transitions has been provided by Slonczewski and Thomas [89]. Such anomalies come from the terms in *G* containing *σ*. The steps of [11,54,57] are followed in order to obtain the same general expression for the compliance

(31) $$s_{ij}=\frac{de_{i}}{d\sigma_{j}}$$

specialized to the first three P−σ coupling terms of the expansion of the Gibb’s free energy

(32) $$\begin{array}{ccc} G & = & \frac{\alpha }{2}P^{2}+\frac{\beta }{4}P^{4}-\frac{1}{2}s_{ij}^{0}\sigma_{i}\sigma_{j}-L_{i}\sigma_{i}P-Q_{i}\sigma_{i}P^{2}-\frac{1}{2}R_{ij}\sigma_{i}\sigma_{j}P^{2}\; \end{array}$$

(33) $$\begin{array}{ccc} \alpha & = & \alpha^{\prime }\left(T-T_{C}\right) \end{array}$$

where the summation over the repeated indexes i,j=1−6 is understood.

In order to keep the derivation and final formulas to an easily comprehensible level, the expansion is truncated to the 4th power of *P*, which in addition is considered as a one-dimensional order parameter, e.g., $P=P_{3}$ of the T-FE phase. The coupling terms bilinear in *P* and *σ*, $-L_{i}P\sigma_{i}$, is forbidden by symmetry in the high-temperature symmetric phase, because it changes sign after application of inversion (the vector Pchanges sign but the centrosymmetric stress tensor σdoes not); since *G* must describe the transition from the C-PE to the FE phases, it must be valid also in the centrosymmetric C-PE phase and therefore the Pσ term is forbidden in the expansion describing a FE perovskite. Yet we maintain it because it will be useful later. In order to describe the successive FE structural transitions, higher powers of Pare necessary, which change somewhat the result, for example, a steplike anomaly acquires a *T* dependence in the low-*T* phase, but the main features of the elastic response do not change. The last two terms in Equation (32) renormalize the coefficient of the $P^{2}$ term, so that we can write

$$\begin{array}{ccc} G & = & A\frac{P^{2}}{2}+\frac{\beta }{4}P^{4}-L_{i}\sigma_{i}P-\frac{1}{2}s_{ij}^{0}\sigma_{i}\sigma_{j}\\ A\left(\sigma ,T\right) & = & \alpha -2Q_{i}\sigma_{i}-R_{ij}\sigma_{i}\sigma_{j} \end{array}$$

The equilibrium value of the order parameter, $P_{0}$, or spontaneous polarization, is found from the condition

(34) $$0={\left.\frac{\partial G}{\partial P}\right|}_{P_{0}}=P_{0}\left(A+\beta P_{0}^{2}\right)-L_{i}\sigma_{i}\;$$

and, since we are interested here in the small signal susceptibility, σ→0 and the above condition reduces to $0=P_{0}\left(\alpha +\beta P_{0}^{2}\right)$, whose solutions are $P_{0}=0$ in the PE phase and

(35) $$\;P_{0}^{2}=-\frac{\alpha }{\beta }=\frac{\alpha^{\prime }\left(T_{C}-T\right)}{\beta }\;$$

in the FE phase. This is the simplest case of 2nd order FE transition below $T=T_{C}$, with spontaneous polarization growing as ${\left(T_{C}-T\right)}^{1/2}$.

Now we derive from Equation (32) the expression of strain to be used in Equation (31), remembering the summation over repeated indexes

(36) $$e_{i}=-\frac{\partial G}{\partial \sigma_{i}}=s_{ij}^{0}\sigma_{j}+L_{i}P_{0}+\left(Q_{i}+R_{ij}\sigma_{j}\right)P_{0}^{2}\;.$$

When we insert this expression into Equation (31) we must take into account that $P_{0}$ depends on stress and therefore also include its derivative with respect to *σ*:

(37) $$s_{ij}=\frac{de_{i}}{d\sigma_{j}}=s_{ij}^{0}+\left(L_{i}+2Q_{i}P_{0}\right)\frac{\partial P_{0}}{\partial \sigma_{j}}+R_{ij}P_{0}^{2}$$

The stress response of $P_{0}$ can be found by differentiating Equation (34) with respect to *σ*

$$0=\frac{\partial P_{0}}{\partial \sigma_{j}}\left[A+\beta P_{0}^{2}\right]+P_{0}\left(\frac{\partial A}{\partial \sigma_{j}}+2\beta P_{0}\frac{\partial P_{0}}{\partial \sigma_{j}}\right)-L_{j}\;,$$

taking the limit σ→0, so that A≃α and $\frac{\partial A}{\partial \sigma_{j}}≃-2Q_{j}$ and solving with respect to $\frac{\partial P_{0}}{\partial \sigma_{j}}$

(38) $$\begin{array}{ccc} \frac{\partial P_{0}}{\partial \sigma_{j}} & = & \frac{L_{j}+2Q_{j}P_{0}}{\alpha +3\beta P_{0}^{2}} \end{array}$$

(39) $$\begin{array}{ccc} & = & \frac{L_{j}+2Q_{j}P_{0}}{2\beta P_{0}^{2}}\;\;\;\left(T<T_{C}\right) \end{array}$$

and finally, for $T<T_{C}$

$$s_{ij}\left(T<T_{C}\right)=s_{ij}^{0}+\frac{L_{i}L_{j}}{2\beta P_{0}^{2}}+\frac{\left(2L_{i}Q_{j}+2L_{j}Q_{i}\right)}{2\beta P_{0}}+\frac{2Q_{i}Q_{j}}{\beta }+R_{ij}P_{0}^{2}$$

with $P_{0}$ given by Equation (35), or

(40) $$s_{ij}\left(T<T_{C}\right)=s_{ij}^{0}+\frac{L_{i}L_{j}}{2\alpha^{\prime }\left(T_{C}-T\right)}+\frac{\left(L_{i}Q_{j}+L_{j}Q_{i}\right)}{\sqrt{\alpha^{\prime }\beta \left(T_{C}-T\right)}}+\frac{2Q_{i}Q_{j}}{\beta }+R_{ij}\frac{\alpha^{\prime }\left(T_{C}-T\right)}{\beta }$$

that corresponds to Equation (5.47) of [54], except for the omission of a coupling term $b_{ij}\sigma_{i}\sigma_{j}P$ in Equation (32), that does not seem relevant here. In the absence of the bilinear coupling term LσP, above $T_{C}$ there is no renormalization of the compliance, $s_{ij}\left(T>T_{C}\right)=s_{ij}^{0}$, because it is $P_{0}=\frac{\partial P_{0}}{\partial \sigma_{j}}=0$ in Equation (36).

The last term from biquadratic stress-polarization coupling, always allowed by symmetry, simply renormalizes the elastic compliance as $s_{ij}^{0}\to s_{ij}^{0}+R_{ij}P_{0}^{2}$, therefore causing a softening or stiffening, depending on the sign of the coefficient *R*, that is linear in *T* or $\propto P_{0}^{2}$ below $T_{C}$. The fourth term, from the linear-quadratic coupling, is the one generally important and considered in the FE transitions: it causes a constant increase of *s* below $T_{C}$, namely a steplike softening; this is what is usually expected and observed at the PE/FE transition.

The bilinear coupling coefficients *L* should be null by symmetry in a cubic perovskite, as explained above, but they are interesting because they cause a major elastic response of the Curie-Weiss type also above $T_{C}$, and we will see that in a transition like T-FE→M-FE, where the polarization rotates away from the direction of the high temperature phase, the permissible $\sigma_{i}P^{2}$ coupling term can be approximated as a bilinear coupling. When $T>T_{C}$, according to Equation (38) it is $\frac{\partial P_{0}}{\partial \sigma_{j}}=\frac{L_{j}}{\alpha }=\frac{L_{j}}{\alpha^{\prime }\left(T-T_{C}\right)}$ and Equation (37) becomes

(41) $$s_{ij}\left(T>T_{C}\right)=s_{ij}^{0}+L_{i}\frac{\partial P_{0}}{\partial \sigma_{j}}=s_{ij}^{0}+\frac{L_{i}L_{j}}{\alpha^{\prime }\left(T-T_{C}\right)}\;.$$

Therefore, the renormalization of the elastic constant from a coupling term bilinear in *σ* and *P* has exactly the form of the Curie-Weiss peak.

**Figure 6:**
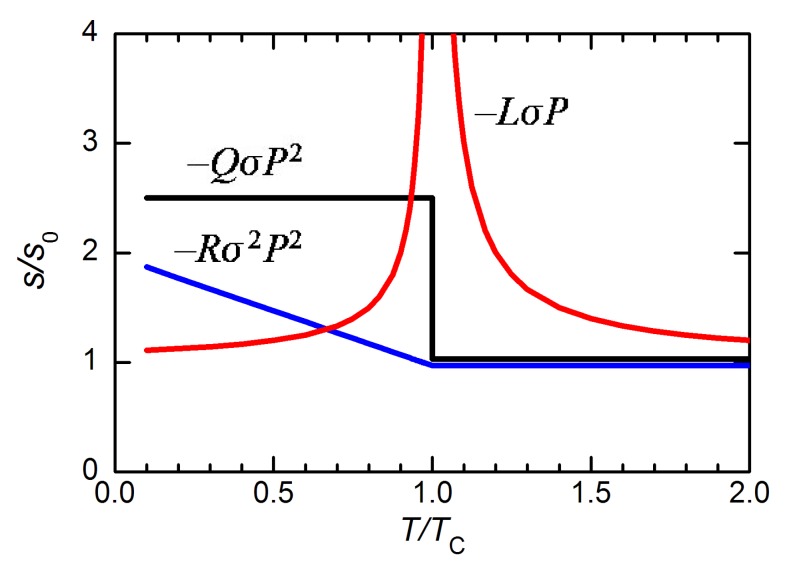
Anomalies in the elastic compliance from the three types of stress-polarization coupling discussed in the text.

These three types of anelastic anomalies are shown in Figure 6.

### 5.2. General case from Landau theory

**Figure 7:**
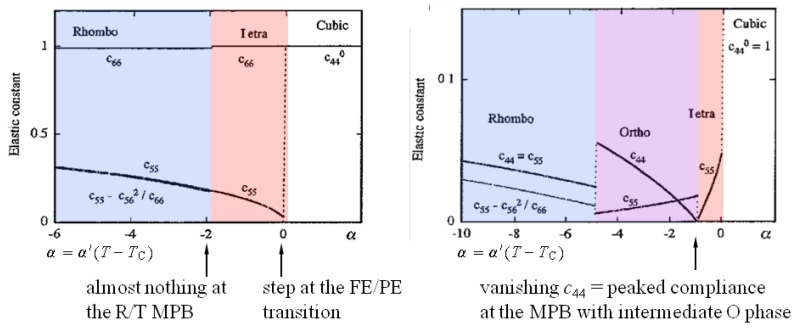
Elastic anomalies at C/T/O/R transitions, using a Landau expansion up to the 6th order in *P* with two sets of coefficients: one reproducing a direct R/T transition and one with the intermediate O phase (adapted from [57] (1999) The Japan Society of Applied Physics).

In the general case that the order parameter P is multicomponent, being a vector, and appears with powers of 6th and higher order, the expressions for the elastic anomalies explicitly written in terms of the expansion coefficients and *T* become soon very cumbersome. Examples can be found in [57], from which Figure 7 is obtained. In that article the expansion is truncated to the 6th order terms, in order to be able to reproduce minima of the free energy in the directions $\langle 100\rangle$, $\langle 111\rangle$ and $\langle 110\rangle$ representing T, R and O FE phases.

Ishibashi and Iwata [57] made numerical simulations of the *T* dependence of the anomalies in the elastic constants in mainly two types of situations: in one the expansion coefficients were chosen in order to have a sequence of C-PE→T-FE→R-FE phases during cooling, in the other an additional O-FE was present between R and T. The interesting result is that the direct R/T transition does not cause almost any effect on the elastic constants, while a peaked softening, up to vanishing of the shear constants $c_{44}$ and $c_{55}$ is found at the O/T border. Two such examples are reproduced in Figure 7, and a similar behavior is found for the dielectric susceptibility [90]. The softening or vanishing of the shear elastic constants, and therefore enhancement or divergence of the shear compliances, is associated with the vanishing of the anisotropy of the free energy up to the 4th order in $P_{i}$. This implies that there is little cost for changing the direction of the polarization, with the associated shear strain, and it is called transverse or rotational instability of the polarization, where “transverse” is with respect to the stable directions in the FE phases.

### 5.3. A Simple Treatment of the Softening With the Intermediate M Phase

**Figure 8:**
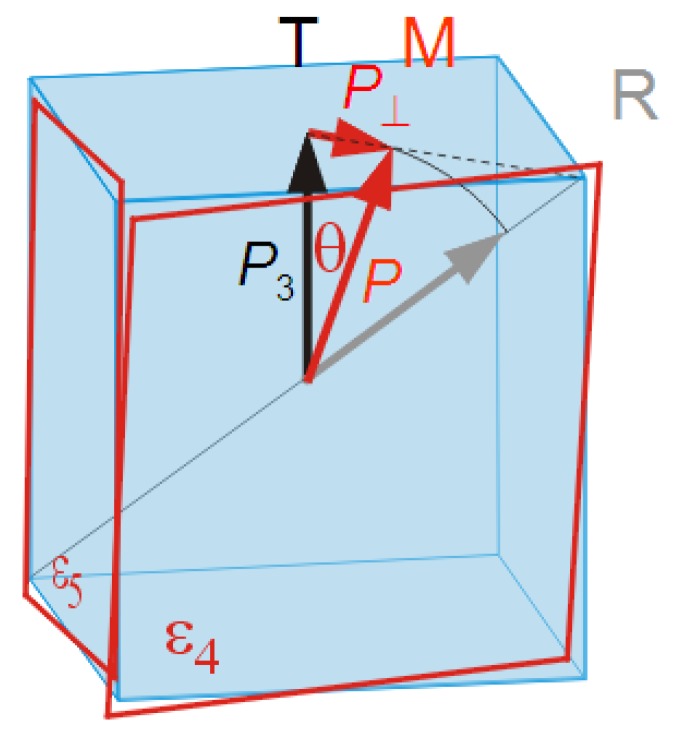
Rotation of the polarization and coupled shear deformation at the T→M transition.

The complexity of the expressions of the elastic constants obtained from the Landau expansion truncated to the 6th order, with the O phase as possible intermediate phase between T and R [57], suggests that the case of the M intermediate phase would be completely intractable, but this is not the case [77,78]. In fact, if we limit ourselves to the case of 2nd order T→M transition occurring at a temperature $T_{0}$, this can be schematized as a rotation of the polarization from P= $\left(0,0,P_{0}\right)$ in the T phase toward $\left(\frac{P_{⊥}}{\sqrt{2}},\frac{P_{⊥}}{\sqrt{2}},P_{0}\right)$ in the M${}_{A}$ phase, as shown in Figure 8, where the transverse polarization $P_{⊥}$ acts as order parameter of the transition.

In this case, the dominant coupling term permitted by symmetry, $G_{c}=-Q_{ijk}\sigma_{i}P_{j}P_{k}$, can be expanded as

$$-G_{c}=Q_{11}\left[\sigma_{3}P_{0}^{2}+\left(\sigma_{1}+\sigma_{2}\right)P_{⊥}^{2}\right]+Q_{12}\left[\left(\sigma_{1}+\sigma_{2}\right)P_{0}^{2}+2\sigma_{3}P_{⊥}^{2}\right]+Q_{44}\left[\left(\sigma_{4}+\sigma_{5}\right)P_{⊥}P_{0}+\sigma_{6}P_{⊥}^{2}\right]$$

where the Voigt notation is used also for the last pair of indexes jk. If the T→M transition is almost 2nd order, for $T≲T_{0}$ it is $P_{⊥}\ll P_{0}$ and $P_{0}≃$ constant, so that we can neglect the terms quadratic in $P_{⊥}$, consider as constants those quadratic in $P_{0}$ and we remain with

(42) $$G_{c}≃-Q_{44}\left(\sigma_{4}+\sigma_{5}\right)P_{⊥}P_{0}\;.$$

that is linear both in the shear stresses $\sigma_{4}$ and $\sigma_{5}$ and in the order parameter $P_{⊥}$. The approximation is valid also for $T\gg T_{0}$ because in the T phase the average value of $P_{⊥}$ is null and its fluctuation are small. In other words, at a transition between two FE states whose polarizations differ in direction but little in magnitude, the linear-quadratic stress-polarization coupling becomes bilinear with respect to the order parameter of the transition, that is the transverse component $P_{⊥}$. Reminding the fact that such term $-L_{i}\sigma_{i}P$ in Equation (32) causes a Curie-Weiss divergence in the corresponding compliances, Equations (40) and (41), we conclude that at the T→M transition the $s_{44}$ and $s_{55}$ compliances are expected to be strongly peaked; in a first approximation as

(43) $$s_{44}=s_{55}=s_{44}^{0}+\frac{Q_{44}^{2}P_{0}^{2}}{\alpha^{\prime }}\left\{\begin{array}{c} \frac{1}{T-T_{0}}\;\left(T>T_{0}\right)\\ \frac{1}{2}\frac{1}{T_{0}-T}\;\left(T<T_{0}\right) \end{array}\right.$$

corresponding to the red curve in Figure 6. Such an ideal divergence is smeared by several factors: *(i)* it is derived in an approximated manner; *(ii)* the transition at the MPB has some first order character; *(iii)* in a ceramic one measures a combination of elastic constants $c_{ij}={\left(s^{-1}\right)}_{ij}$, of which only $c_{44}=1/s_{44}$ and $c_{55}=1/s_{55}$ ideally vanish at the MPB, while the others do not. Figure 8 shows the shear strains associated with the enhanced compliance, $e_{4}=s_{44}\sigma_{4}$ and $e_{5}=s_{55}\sigma_{5}$.

### 5.4. Thermal Fluctuations

The Landau theory neglects the role of fluctuations, which are important in the close vicinity to a 2nd order transition, and produce frequency dependent peaks in the susceptibilities and precursor softenings in the high temperature phase [91,92,93,94,95]. In principle, their role might also become important near the MPBs, where the free energy becomes particularly flat with respect to certain polarization modes (mainly rotations). Another case in which fluctuations are important is that of relaxors, where the polar nanoregions (PNR) that start forming below the Burns temperature, represent a sort of larger and longer lived type of fluctuations with respect to those in a classical PE phase. Interestingly, the best piezoelectric performances are found in solid solutions of PbTiO${}_{3}$ with relaxors (see Section 9), and an atomistic model for this has been proposed [96] and experimentally verified, though only in the PE phase [97]. It has also been proposed that in solid solution with relaxors the giant electromechanical response can be viewed as a critical phenomenon [98].

In relaxors, the PNR, therefore P, interact with stress thanks to the electrostrictive coupling $G_{c}$, Equation (27) and produce softening [99,100,101]. This can be seen using the Slonczewski and Thomas [89] derivation of the elastic anomaly at a phase transition, that is alternative to Section 5.1 but gives the same result. The contribution of the OP $P_{i}$ with susceptibility $\chi_{ij}={\left(\partial^{2}F/\partial P_{i}\partial P_{j}\right)}^{-1}$ to the elastic stiffness $c_{ij}$ can in general be written as [89]

$$c_{ij}=c_{ij}^{0}-\frac{\partial^{2}F_{c}}{\partial P_{k}\partial e_{i}}\chi_{kl}\frac{\partial^{2}F_{c}}{\partial P_{l}\partial e_{j}}$$

where the coupling term $F_{c}$ in the Helmholtz free energy *F* is $F_{c}=g_{ijk}e_{i}P_{j}P_{k}$ (compare with $G_{c}$ in Equation (27)). Following [100], in a simple longitudinal case with negligible contribution in the sum from terms with mixed indexes, hence i=j=k=3 and $g_{333}=g\gg g_{331}$, one gets c= $c^{0}-4\chi g^{2}P^{2}$ which is the same as Equation (40) with L=R=0 for $T<T_{C}$, while above $T_{C}$ one can substitute $P^{2}$ with $\langle P^{2}\rangle =\langle \delta P^{2}\rangle$ from fluctuations or PNR:

(44) $$c=c^{0}-4\chi g^{2}\langle \delta P^{2}\rangle \;.$$

This type of softening is proportional to the mean square fluctuation of the polarization, and therefore is significant in the PE phase, but much less in a FE phase. In fact, in the FE phase the fluctuations δP are on top of the spontaneous polarization, and should have an exceptionally large magnitude in order to make a significant contribution, or may contribute to an otherwise small transverse susceptibility, as calculated for the dielectric $\chi_{⊥}$ [56]. Yet, the case of polarization unstable against rotations also produces the approximately bilinear coupling with the shear strain and stress, as shown in the previous Section, and hence the divergence in the shear elastic compliance of the Curie-Weiss type, so that it does not seem necessary to analyze the effect of fluctuations on the compliance.

Actually, it will be shown that in the reference case of PZT, at compositions where the enhancements of the piezoelectric susceptibility and elastic compliance are maximum, the MPB has first order character with thermal hysteresis of the susceptibilities between heating and cooling. As also remarked by Khachaturyan [56], the thermal hysteresis is due to the partial freezing of the metastable phase when the new phase is more stable, and therefore excludes that thermal fluctuations play a determinant role. Still, in cases with little thermal hysteresis, hence close to 2nd order, and even more at tricritical points, the role of fluctuations should be carefully considered.

## 6. Intrinsic and Extrinsic Contributions to the Piezoelectric Effect

Up to now we have dealt with the susceptibilities of single domains, or intrinsic susceptibilities, representing the case where the polarization has the same direction over all the crystal, and it can only uniformly change in magnitude or rotate away from the initial state under an applied field, possibly switching to another crystallographically equivalent direction. In ceramic materials, however, the electrostatic and elastic energies are minimized by the formation of several domains having different orientations of the polarization and spontaneous strain, separated by domain walls (DW). For ferroelastics, when the two domains are mirrored through the wall, this is also called twin wall. The same in ferroelectrics, with the possibility of inversion of the polarization in one of the domains, which does not change the strain but allows the configuration of the polarization to become the uncharged head-to-tail. In this case, the overall polarization and strain may change under application of an external field through the motion of the walls, that expand the domains with lower energy in the field at the expenses of the domains with higher energy. The additional response is the extrinsic contribution to the susceptibilities from DW, and may represent a considerable fraction of the overall response. The DW motion generally requires an energy that is smaller than the anisotropy energy of a uniform rotation of P. Therefore the response to a field has an important contribution from DW motion, unless one has a monodomain single crystal [102,103,104] or a crystal suitably oriented and polarized in order that all the domains remain equivalent under the application of the field [105]. Additional extrinsic contributions may come from the motion of point or extended defects, as dielectric and anelastic relaxation processes. Notice also that when a point or extended defect has both stress and electric active coordinates, such as an electric dipole with tetragonal axis or a $90^{{}^{\circ}}$ FE DW, anelastic and dielectric relaxations are accompanied by the piezoelectric relaxation, as shown in Section 2.1.

The dynamics of DW is generally nonlinear and quite complicated. Theoretical estimates of the contributions of DW to the various susceptibilities of tetragonal BaTiO${}_{3}$ have been proposed by Arlt and coworkers [106], neglecting inertia and dissipative effects. The DW dynamics is often studied by strain−E field loops and quantitatively interpreted in the framework of the Rayleigh equations, so allowing intrinsic and extrinsic effects to be distinguished [25,107,108]. In this manner, for example, it has been established that in PMN-PT around the MPB, with piezoelectric response above 2000 pC/N, the extrinsic contribution is <10% [107].

The importance of the extrinsic contributions to the susceptibilities certainly increases with increasing the amplitudes of the fields and strains involved in the applications or experiments. On the other hand, only the linear anelastic response from the reversible motion of DW is observed when measuring the Young’s modulus in resonant experiments, due to the low amplitude and relatively high frequency, and there is hardly any contribution from DW in Brillouin experiments, at frequencies of tens of GHz. Instead, the nonlinear response of DW can be studied with the DMA at various levels of static force. In this manner, it has been found that the Young’s modulus of commercial PZT follows Rayleigh’s law, while the dependence of the anelastic spectrum of PMN-PT on bias was too small for this type of analysis [109].

Another method to separate extrinsic from intrinsic piezoelectric response is the comparison between ceramics and monodomain or domain engineered crystals, when they are available. In this manner it has been shown that in (PMN)${}_{0.67}($PT)${}_{0.33}$, at the middle of the R/T MPB, the piezoelectric response is >80% due to the intrinsic giant $d_{15}$ [103,104]. Similarly, no enhancement of the piezoelectric properties has been found in “domain engineered” KNbO${}_{3}$ crystals with respect to the single domain ones [110]. These results are strong indications that an explanation only in terms of adaptive phase cannot account for the observed enhancements of the piezoelectric coefficients at the MPB, even at high fields.

Recently, the progress in the neutron diffraction and synchrotron XRD techniques, made it possible to realize time resolved experiments where the microscopic strain response to an alternate field is followed, by monitoring selected diffraction peaks. In a neutron diffraction experiment on ${\left(\text{PbTi}\right)}_{0.64}{\left(\text{BiSc}\right)}_{0.36}$O${}_{3}$ the intrinsic frequency independent response to an alternate electric field and extrinsic frequency dependent responses where measured between 0.01 and 1 Hz, finding a strong extrinsic contribution from the M phase using field amplitudes less than half of the coercive field [111].

It is also possible that the nonlinear effects associated with strains and electric fields at DW enhance the piezoelectric effect, independently of the DW motion [112].

## 7. Monoclinic Phases, Polarization Rotation and Enhanced Piezoelectric Response

The topic of the enhanced piezoelectric response at MPBs is reviewed in various articles and books cited in the Introduction. In this Section additional background information is provided, in order to better put in context the results on the elastic properties.

### 7.1. Poling Induced Monoclinic Phase

The flatness of the free energy of certain PT-based solid solutions with respect to the polarization direction, implies an easiness in stabilizing the M phase through composition or external fields. Guo *et al.* [113] proved that poling in the MPB region does not simply change the population of R and T domains, as implied by the original statistical model where the piezoelectric effect is related to the number of possible directions for P, but induce a permanent change in the unit cell. To this end, they performed synchrotron XRD experiments with *in situ* application of a poling electric field, and demonstrated that the rotation of the polar axis in the monoclinic plane may cause a M distortion of the originally R or T cells. In other words, poling may stabilize the M phase in the otherwise T or R phase. This process has also been shown to result in dielectric anomalies with frequency dispersion of the Vogel-Fulcher type in PZT 52/48 [113,114]

### 7.2. Intermediate Phase Induced by Disorder and Domain Strain Accommodation

A possible mechanism for the stabilization of M phases, though not homogeneous phases, is considered the chemical disorder causing strong local distortions away from the T, R or O FE phases of the end members of the solid solutions. A striking example is K${}_{1-x}$Na${}_{x}$NbO${}_{3}$ (KNN): both NaNbO${}_{3}$ and KNbO${}_{3}$ are O, but around x∼0.5 the structure is M Pm [115]. This phase cannot be considered as bridging two different orientation of the polarization, as usually assumed for the R/M/T case, and has rather been associated with the local lowering of symmetry due to the disorder in bond lengths (∼30%) of Na and K ions with substantially different sizes [116]. Accordingly, in a recent calculation based on a core shell model for the ionic polarization, it was found that, if PbZr${}_{1/2}$Ti${}_{1/2}$O${}_{3}$ is chemically ordered with Ti and Zr alternated along all directions, the structure is tetragonal P4mm, while it is monoclinic if Zr and Ti are randomly distributed [117].

Large internal strain and electric fields do not necessarily need chemical disorder, but can be generated by domain structures, and in fact there are observations of M phases stabilized by domain structures near certain phase transformations. A notable example is BaTiO${}_{3}$, totally ordered from the chemical point of view, at the O/T border [118,119,120,121]. The phenomenon of the stabilization of a M phase by domain structures can also be reproduced with phase field simulations [22].

### 7.3. The Monoclinic Phase as Ground State of PbTiO${}_{3}$-Based Solid Solutions

As explained more at length in Section 9, PbTiO${}_{3}$ (PT) forms MPBs with M phases in solid solution with PbZrO${}_{3}$ [122], Pb(Mg${}_{1/3}$Nb${}_{2/3}$)O${}_{3}$ (PMN) [123], PbZn${}_{1/3}$Nb${}_{2/3}$O${}_{3}$ (PZN), Bi(Ni${}_{1/2}$Ti${}_{1/2}$)O${}_{3}$ [124] and Bi(Mg${}_{1/2}$Ti${}_{1/2}$)O${}_{3}$ [125], but not all these phases have the same space group.

**Figure 9:**
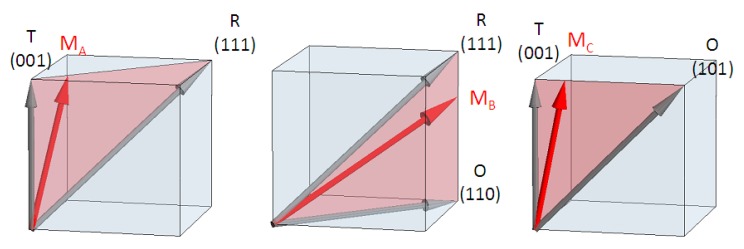
Direction of the spontaneous polarization in the three types of monoclinic phases with the usual nomenclature M${}_{A}$, M${}_{B}$ (Cm) and M${}_{C}$ (Pm) introduced in [58] (see also e.g., [126]).

Following the nomenclature introduced by Vanderbilt and Cohen [58,123], the M phases are indicated as M${}_{A}$, M${}_{B}$ and M${}_{C}$ according to the direction of P (Figure 9): M${}_{A}$, with space group Cm and where the direction of P is intermediate between the R and T phases, M${}_{B}$, with space group Cm intermediate between R and O, and M${}_{C}$, with space group Pm and intermediate between T and O; the latter can be considered as the average of a twinned T phase [127].

As shown in the previous paragraphs, if the free energy is almost isotropic with respect to the direction of P, small external fields or compositional fluctuations, or possibly even internal strains from DWs, may easily stabilize a M phase from a T or R ground state. Yet, there is theoretical and experimental evidence that the M phase may be the ground state of PT related materials. Ahart *et al.* [67] showed that the pressure-*T* phase diagram of PbTiO${}_{3}$ presents two MPBs between T, M and R phases with increasing *p*. The M phase is further split into M${}_{C}$ and M${}_{A}$, with increasing *p*, but is the ground state in homogeneous PbTiO${}_{3}$ and is not the result of nanotwinning or compositional heterogeneity. The M state would arise when the conditions are intermediate between the two extreme cases of stable minima of the polarization along $\langle 100\rangle$ and along $\langle 111\rangle$. The latter case is characteristic for example of the R phase of BaTiO${}_{3}$, while the $\langle 100\rangle$ direction is stabilized by the large tetragonal strain of PbTiO${}_{3}$ at ambient conditions, due to the electronic configuration of Pb${}^{2+}$ (the so-called nonsymmetrically disposed lone pair). Increasing *p* reduces the tetragonal strain, gradually making the $\langle 111\rangle$ minima the stable ones, and one passes through the intermediate M phases. The MPB between M and T phases in PT-based solid solutions, therefore, would result from a tuning to the ambient condition of the conditions for the MPB *vs.* *p* in pure PbTiO${}_{3}$ through chemical pressure [67,128].

It must be said that even the apparently simple case of PbTiO${}_{3}$, and hence the above interpretation of the MPB in PT-based perovskites, is quite controversial, because both the first principle calculations [66] and the experiments [67] finding stable M phases at high pressure have been questioned and other interpretations have been proposed [129,130]. Not only the evidence on the ease of polarization rotation has been challenged [130], but also the assignment of the structural phases under pressure [129,130]. In particular octahedral tilting, which was not considered in [67], was found to play a determinant role in accommodating the mismatching bond lengths at high pressure.

### 7.4. Monoclinic Phase = High Piezoelectric Coefficients?

It is contended [131] that a M phase is not a sufficient condition for large piezoelectric strains, that are ultimately due to a large elastic compliance, and when the piezoelectric strain is exceptionally high, they can change a R or T structure into M.

A monoclinic phase coexisting with a T phase certainly implies that P may change direction upon application of an external field with ease, but large piezoelectric coefficients result also from the magnitude of the ferroelastic strain. It is therefore understandable that NBT, though recently recognized to have M structure at room temperature, has a small piezoelectric coefficient [132]; in fact the distortions of NBT from the parent cubic phase are much smaller than in Pb or Ba based perovskites.

### 7.5. Tricritical Points Are Better Than MPBs?

Throughout this article and in the cited literature it has been stressed how the flattening of the free energy with respect to the polarization rotation and/or extension enhances the piezoelectric coupling, because it gives rise to a rotational and/or extensional instability in the spontaneous polarization, which in turn enhances both the dielectric and elastic susceptibilities, both contributing to the piezoelectric effect as appears from Equation (4). It would therefore seem that an even higher piezoelectric effect should be found near the tricritical point where the MPB meets the $T_{C}\left(x\right)$ border. In that region of the phase diagram the free energy should be extremely flat, allowing instability of both magnitude and direction of P. Indeed, there is a considerable number of articles devoted to this topic, but the experimental evidence is that the maximum values of the piezoelectric coefficients d are generally not for longitudinal $d_{33}$, due to change in magnitude of P, but in the shear or transverse $d_{15}$ or $d_{31}$, due to the rotation of P and not close to $T_{C}$. This phenomenon can be better understood by considering Equation (29), where it is made clear that the piezoelectric effect can be seen as piezostrictive effect, $e=QP^{2}$, biased by the spontaneous polarization *P*, which moves the response away from the minimum of the parabola at P=0 to the point with de/dP= 2QP. Therefore, also a large magnitude of *P* is required in order to have a large *d*, and this is not the case near $T_{C}$, where *P* vanishes in the PE phase. The FE/FE transition has the advantage over the FE/PE transition of large *P* in both phases, and the enhancement of the susceptibilities comes from the transverse instability. This fact has been especially emphasized in recent studies on BCTZ [133,134] (Section 11).

## 8. PZT

In what follows the notation PZT−100x will be used for PbZr${}_{1-x}$Ti${}_{x}$O${}_{3}$.

### 8.1. Measurements With the Torsion Pendulum

**Figure 10:**
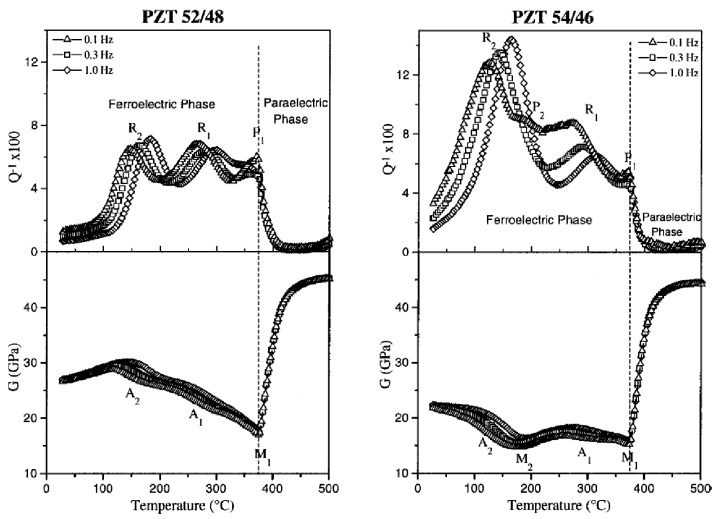
Shear modulus *G* and elastic energy loss coefficient $Q^{-1}$ of PZT-48 and PZT-46 measured at 0.1, 0.3 and 1 Hz. (from Ref. [135], Copyright American Institute of Physics).

The group of Fantozzi carried out various measurements on ferroelectric ceramics with the torsion pendulum. Figure 10 presents the anelastic spectra of PZT-46 and PZT-48 measured between 0.1 and 1 Hz [135,136].

Besides the expected step-like softening at $T_{C}$, labeled M${}_{1}$, the interesting features in the present context are the minimum labeled M${}_{2}$ and the thermally activated relaxation processes visible in the losses, labeled R${}_{1}$ and R${}_{2}$. The minimum M${}_{2}$, whose position is almost independent of frequency, was recognized as the T/R transformation, since it occurs at $T_{\mathrm{MPB}}$. In PZT-48 it is $T_{\mathrm{MPB}}≃250$ K, and the minimum is below the experimental temperature range, but the hight−T side of the softening is evident. The relaxation processes show clear shifts to higher temperature when measured at higher frequency, as in Figure 1, and have been attributed to the motion of DW interacting with charged point defects, namely O vacancies. We are not interested here in the detailed mechanisms behind these relaxation processes, but they clearly involve DW and two observations are important: they are well visible in the losses, but their contribution to the softening is comparatively weaker than the anomalies due to the phase transformations, M${}_{1}$ and M${}_{2}$. In PZT-46 the peak in the losses is close to the minimum M${}_{2}$, but it only affects its low temperature side; in PZT-48 the losses even become negligible in correspondence to M${}_{2}$, whose minimum is below the minimum temperature of the measurement. It can be concluded that there is a peaked softening in *G* at $T_{\mathrm{MPB}}$, that cannot be due to the motion of DW and therefore is an intrinsic feature of the transition.

### 8.2. Measurements With Flexural Resonance

Other reports have appeared of the elastic properties at frequencies of Hz and kHz of PZT at compositions near the MPB [137,138,139,140], but a systematic anelastic and dielectric study of the whole phase diagram of PZT, with particular care to the MPB region, has been conducted by Cordero and coworkers [76,77,78,80] with the method of the free flexural resonance.

**Figure 11:**
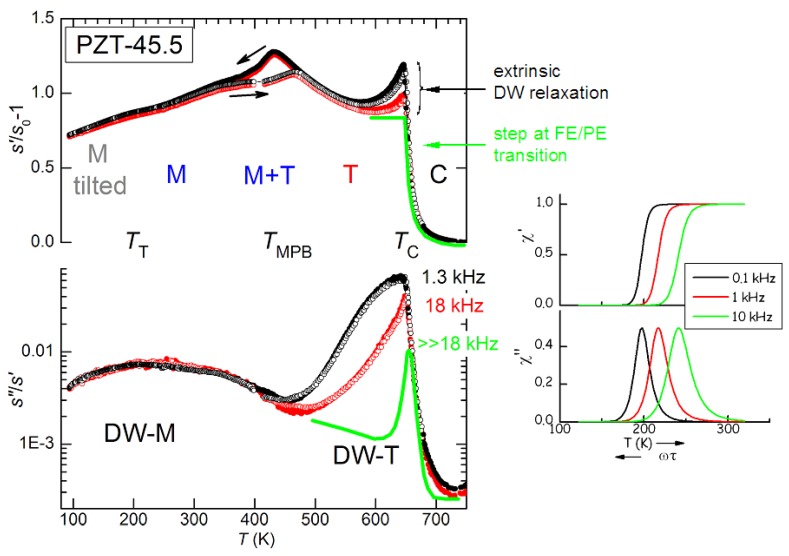
Real part of the compliance s=1/Y and elastic energy loss coefficient of PZT-45.5 measured at 1.3 and 18 kHz (data from Ref. [77]). The hypothetical green curves are for a much higher frequency, where the tail of the thermally activated DW relaxation is shifted at $T>T_{C}$. On the right is a sketch of a relaxational contribution with its frequency dependence.

Figure 11 presents a typical anelastic measurement during heating and cooling, exciting during each run the 1st and 3rd flexural modes at 1.3 and 18 kHz (in the PE phase). Similarly to the measurements at lower frequency (Figure 10) there is a steplike softening below $T_{C}$, preceded by some precursor softening in the PE phase, that rounds the step. The frequency dispersion just below $T_{C}$ is due to the thermally activated motion of the DW in the T phase; it is accompanied by losses that would be peaked in the PE phase, where the DW do not exist any more, and all these curves are shifted to higher *T* at the higher frequency. This relaxation process certainly corresponds to that measured with the torsion pendulum and labeled R${}_{1}$ in Figure 10. The hypothetical green curves for a much higher frequency show that when the tail of the thermally activated DW relaxation is shifted to $T>T_{C}$, one remains with a step in the compliance, with upper edge at $T_{C}$, and possibly with a spike in the losses due to fluctuations. Therefore, the anelastic spectrum of PZT has the steplike softening at $T_{C}$, due to the $\sigma P^{2}$ coupling, as in Figure 6.

#### 8.2.1. Peaked Softening at $T_{\mathrm{MPB}}$: Intermediate Monoclinic Phase

On the other hand, what is observed at $T_{\mathrm{MPB}}$ is not a step, but a peak, in accordance with M${}_{2}$ in Figure 10 and as expected from a coupling of the type σP in Figure 6. Following the discussion of Section 5.3, we conclude that the transition at $T_{\mathrm{MPB}}$ is not between R and T phases, but between M and T, where the order parameter is $P_{⊥}$ and changes quasicontinuously, so that the linear quadratic coupling term $-Q_{ijk}\sigma_{i}P_{j}P_{k}$ contains terms of the type $-Q_{44}\sigma_{4}P_{⊥}P_{0}$, linear in *σ* and $P_{⊥}$. Actually, the simulations of Ishibashi and Iwata [57] described in Section 5.2 indicate that a transition between O and T phases would have a similar effect, since both cases share a marked isotropization of the free energy with consequent transverse instability of the polarization and of the shear compliance. From anelastic experiments on ceramics it is impossible to tell whether the phase below $T_{\mathrm{MPB}}$ is M or O, but there is abundance of diffraction experiments showing that it is M. Yet, there is certainly no continuous change of $P_{⊥}$ during the transition, because it is clearly of first order. In fact, $s^{\prime }$ exhibits a hysteresis between heating and cooling, that marks the temperature range of M+T coexistence. Inclusions of T domains below $T_{\mathrm{MPB}}$ have also been identified from diffuse X-ray scattering in single crystals [141]. It is important to note that there is no hysteresis at all in the losses, and this demonstrates once more that there is no contribution from DW relaxation to the peaked softening at $T_{\mathrm{MPB}}$; if there were some, the hysteresis would be much more visible in the losses than in the real part, as is the case just below $T_{C}$.

**Figure 12:**
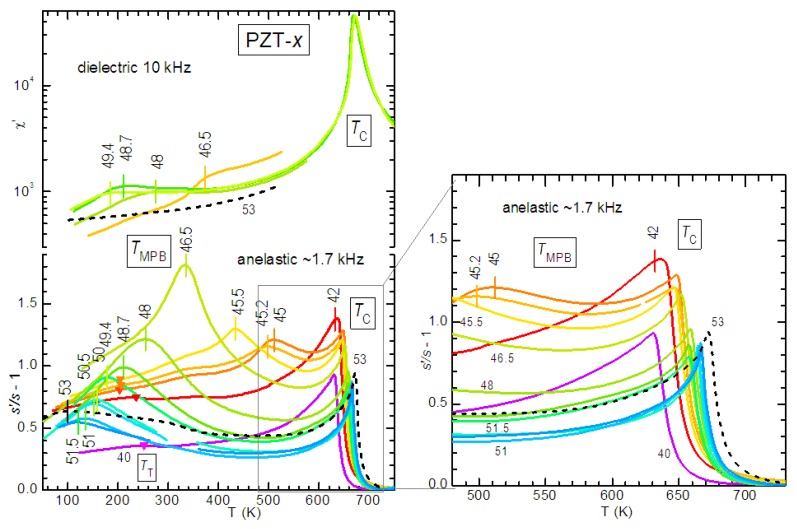
Real part of the compliance s=1/Y (1−2 kHz) and of the dielectric susceptibility (10 kHz) of PZT−x (data from Refs. [76,77]). The compositions x=%Ti are indicated next to each curve. The vertical bars indicate $T_{C}$ and $T_{\mathrm{MPB}}$ while the triangles $T_{T}$. On the right is a detail of the region around $T_{C}$.

Figure 12 shows the real parts of the reciprocal Young’s modulus $s^{\prime }$ and dielectric susceptibility $\chi^{\prime }$ measured at several Ti compositions 0.4≤ x≤ 0.53 [76,77,78]. Also $\chi^{\prime }$ is peaked at $T_{\mathrm{MPB}}$, but comparatively much less than at $T_{C}$. On the contrary, at compositions near the middle of the MPB line, x≃0.465, the peak of the compliance has its maximum value and is comparable if not larger than the estimated intrinsic softening below $T_{C}$. All this is in agreement with the phenomenology described in Section 5.2 and Section 5.3, when the free energy becomes isotropic enough to have a thermodynamically stable intermediate M or O phase. The lack of any sign of enhanced contribution of DW to the susceptibilities at $T_{\mathrm{MPB}}$ does not exclude the occurrence of nanotwinning or domain miniaturization, but simply that their role is not relevant at the low fields and the frequencies involved in these measurements.

The temperature $T_{\mathrm{MPB}}$ is chosen as the maximum of $s^{\prime }$ and when plotted in the x−T phase diagram (Figure 5) demonstrates a good correlation with the MPB line, $T_{\mathrm{RT}}+T_{\mathrm{MT}}$, from diffraction [8,83] and other experiments, including Raman spectroscopy [82], dielectric [81] and infrared reflectivity [84]. There are however two differences. The first, a feature that is overlooked in the majority of the articles except Refs. [76,142], is the bending of the MPB line toward low *x* before it meets the $T_{C}$ line. This feature is well attested in other solid solutions of PT with relaxors (see Section 9.1), and also reproduced in phase diagrams obtained from a Landau free energy that combines the OP of the two end members [74] (Figure 5). In the anelastic data it is attested by the $s^{\prime }\left(T\right)$ curve of PZT−42, that presents a shoulder just below $T_{C}$ at $T_{\mathrm{MPB}}≃632$ K, instead of the plain decrease below $T_{C}$ from the tail of the DW relaxation observed at all the other compositions. This feature is better seen in the enlargement in Figure 12.

#### 8.2.2. Octahedral Tilting and the R/M Border

The other difference with respect to the generally accepted MPB line straight down to 0 K is an upward bending before meeting the $T_{T}\left(x\right)$ line of the octahedral tilt instability. The anelastic spectra of PZT revealed many new features, mostly related to the tilting of the octahedra in the regions of the two MPBs between R-FE and T-FE and between O-AFE and R-FE phases [76,77,78,143]. These features have no direct impact on the topic treated here, except perhaps on the issue of the R/M border and therefore they are treated briefly. The new $T_{\mathrm{IT}}$ line appearing in Figure 5, up to now revealed only by a step in the compliance, is interpreted as the onset of disordered or uncorrelated octahedral tilting, while long range order that is attained below $T_{T}$. Octahedral tilting is generally considered as independent or in competition with the FE order [144,145], but the series of anelastic spectra seem to indicate that the $T_{\mathrm{MPB}}\left(x\right)$ and $T_{T}\left(x\right)$ lines are not independent of each other and, instead of crossing they merge or remain close to each other in the high *x* end. This fact, together with the observation that also the $T_{\mathrm{IT}}\left(x\right)$ merges with $T_{C}\left(x\right)$ [76], suggest that the coupling between tilting, described by the rotation angle *ϕ* of the octahedra, and P may be cooperative rather than competitive, namely a mixed term in the free energy of the type $-R\phi^{2}P^{2}$ [76,80] (see Equation (32)). The merging of $T_{\mathrm{MPB}}$ and $T_{T}$ with possible mixing of the antiferrodistortive and FE degrees of freedom occurs well below room temperature, and hence it does not seem to be of interest when dealing with piezoelectricity at higher temperatures. Instead, it may be of interest the observation that the shape of the anomaly at $T_{T}$ changes within the range 0.465< x< 0.48, possibly indicating the presence of a nearly vertical R/M boundary [76].

**Figure 13:**
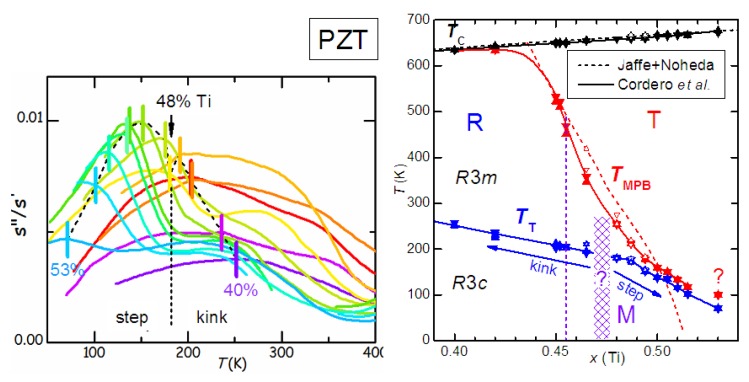
Elastic energy loss of PZT−x measured together with $s^{\prime }$ shown in Figure 12 (data from Refs. [76,77]). The vertical bars indicate $T_{T}$ and are plotted in the detail of the MPB region of the phase diagram.

In Ti-rich samples, the step in $s^{\prime }$ at $T_{T}$ becomes less and less evident, because its amplitude decreases and it is masked by the peak at $T_{\mathrm{MPB}}$, but the anomaly remains evident in the elastic energy loss. This is shown in Figure 13, where the $s^{\prime \prime }/s^{\prime }$ curves are those corresponding to the $s^{\prime }$ curves in Figure 12. The vertical bars at $T_{T}$ are joined by a dashed line, in order to show that there is a regular trend with varying *x*, with a change between 0.465< x< 0.48, where the anomaly transforms from kink to step. Something is clearly occurring around that composition, indicated as a dashed area in the phase diagram in the same figure, and the first thing that comes to mind is a border between R and M phases. Such a border is set at the slightly lower composition x=0.455 by Noheda [8] (dashed line) and tentatively suppressed by Glazer *et al.* [146] after the observation of diffuse scattering in the R phase, that indicates locally lowered symmetry from partially correlated Pb displacements. According to this view, there is no R/M border because the R and M phases are both monoclinic, but with gradually varying degree of correlation of the (mainly) Pb displacements.

### 8.3. Brillouin Scattering: Reduced Amplitude of the Anomaly at $T_{\mathrm{MPB}}$ in $s_{11}$

**Figure 14:**
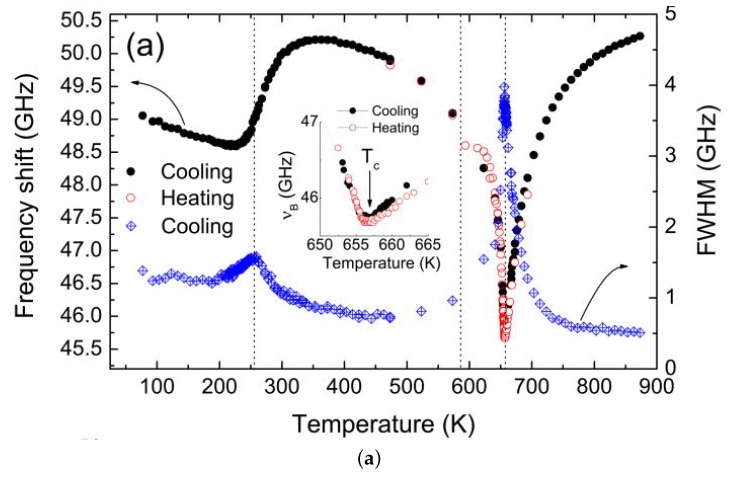

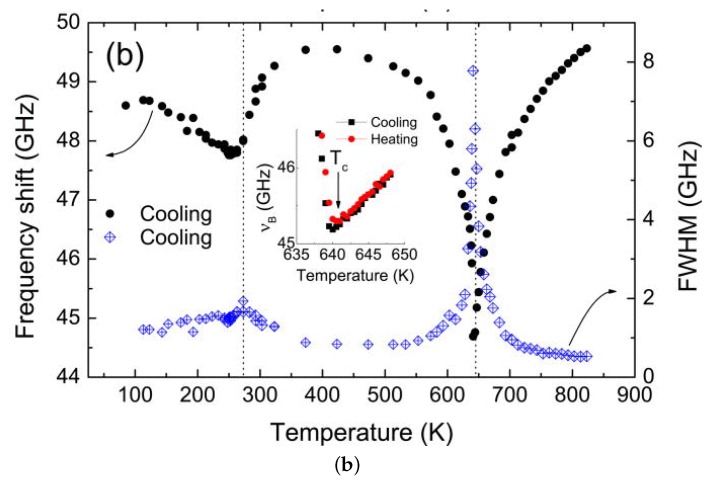
The temperature dependences of the Brillouin frequency shift and the FWHM of the LA mode, corresponding to $c_{11},$ for (**a**) PZT-45; and (**b**) PZT-42. The insets show the extended view of the minimum of the Brillouin frequency shift of each sample. (from Ref. [53], Copyright American Institute of Physics).

The anelastic spectrum of PZT has been measured also at tens of GHz by Brillouin scattering on single crystals with x=0.42 and 0.45 [53] (Figure 14). The Brillouin shift of the LA mode along the $\left(100\right)$ direction corresponds to the elastic constant $c_{11}$, which is also $1/s_{11}$ in the cubic phase.

At these frequencies the anomaly at $T_{C}$ becomes a sharp peak rather than a step, accompanied by an equally sharp peak in the losses (the full-width at half maximum of the scattered line). This peaked anomaly has to be associated with a contribution from fast fluctuations with amplitude ∝ω, and therefore hardly visible in the kHz range but preponderant in the GHz range. The step near 270 K is associated with octahedral tilting, and is more evident in $c_{11}$ at GHz than in the polycrystalline Young’s modulus at kHz. Instead, the prominent peaked softening observed in the Young’s modulus at $T_{\mathrm{MPB}}$ becomes a small step at ∼590K for x=0.45, superimposed to the sharp dip at $T_{C}$. This constitutes a nice confirmation that the softening in the Young’s modulus at the MPB of ceramic PZT is indeed due to $s_{44}$ and $s_{55}$ and not to $s_{11}$. The step in $c_{11}$ is compatible with the linear-quadratic coupling term $\sigma_{i}P_{i}^{2}$ (i=1−3), while the peaked softening in the remaining shear elastic constants with the quasi-bilinear term Equation (42) is possible at a M/T phase transition. The combination of the two sets of data therefore proves the purely transverse nature of the instability at the MPB of PZT.

## 9. Other PT-Based Solid Solutions

### 9.1. Monoclinic Phases and Rotation of the Polarization Under An Electric Field

The materials that can exhibit the highest piezoelectric coefficients are solid solutions of PbTiO${}_{3}$ with Pb(Mg${}_{1/3}$Nb${}_{2/3}$)O${}_{3}$ (PMN) and Pb(Zn${}_{1/3}$Nb${}_{2/3}$)O${}_{3}$ (PZN), and also more complicated solid solutions are being studied. While part of the confusion on the nature of the MPB in PZT may be due to the difficulty of obtaining single crystals, PMN-PT and PZN-PT can be grown as large single crystals, that can be up to five times more piezoelectric than ceramic PZT; nonetheless, the consensus on the nature of the MPB is not better than for PZT. This is likely due to the fact that, while in PZT the chemical disorder on the Ti sublattice is essentially reduced to disorder in the Ti${}^{4+}$ and Zr${}^{4+}$ ion sizes, in PMN and PZN the additional charge disorder between Nb${}^{5+}$ and Mg${}^{2+}$ or Zn${}^{2+}$ makes the materials relaxors, with the additional phenomena of polar nanoregions (PNR) below the Burns temperature, their freezing, and peculiar features of the phonon dispersions [14,147,148]. All these features make the solid solutions of these relaxors with PbTiO${}_{3}$ even less amenable to clear-cut characterizations than PZT.

**Figure 15:**
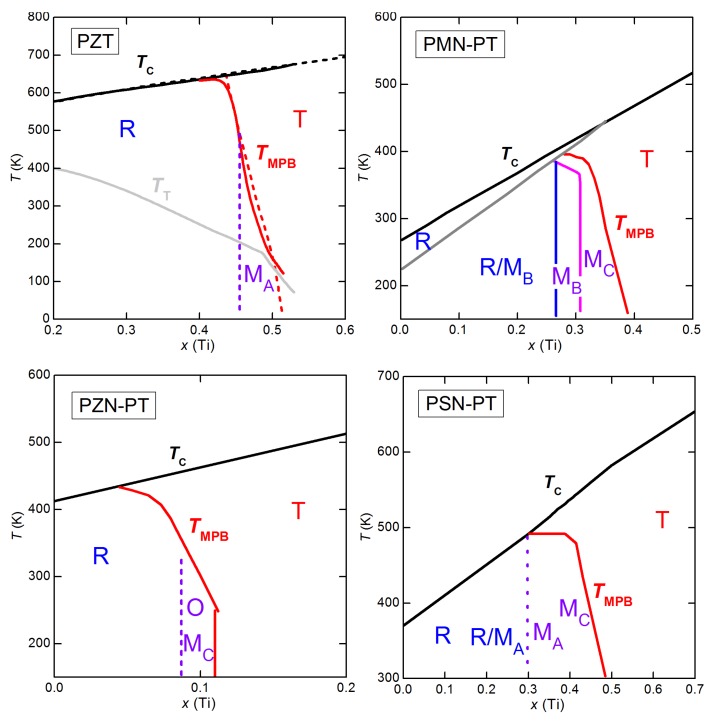
Phase diagrams of four solid solutions of PbTiO${}_{3}$ (PT) with antiferroelectric PZ and relaxor Pb(Mg${}_{1/3}$Nb${}_{2/3}$)O${}_{3}$ (PMN), PbZn${}_{1/3}$Nb${}_{2/3}$O${}_{3}$ (PZN) and PS. PZT: the continuous and dashed lines are, as in Figure 5, from our anelastic and dielectric data and from [8], respectively; PMN-PT: from Ref. [126]; PZN-PT from [8]; PSN-PT from [149].

The literature on these solid solutions is vast, and only few aspects related to the main topics in this article are touched upon here. Figure 15 presents the MPB regions of the phase diagrams of three solid solutions of PbTiO${}_{3}$ with three relaxors, together with that of PZT. According to the initial works of Noheda [8], PZN-PT has an intermediate O or M${}_{C}$ region at the MPB between R and T phases, and PMN-PT an intermediate M${}_{C}$ phase, which differs from the M${}_{A}$ observed in PZT in the direction of the polarization. As shown in Figure 9, the M${}_{A}$ phase is intermediate between the T and R, while the M${}_{C}$ between T and O [8,126].

A subsequent study [126] puts in evidence anomalously broad diffraction peaks of the R phase of PMN-PT, and assigned them to local M${}_{B}$ symmetry mainly arising from disordered displacements of Pb away from the average $\left(111\right)$ direction, similarly to what had been proposed for PZT [146,150]. The additional M${}_{B}$ phase, intermediate between R and O (Figure 9), is also introduced in the phase diagram of PMN-PT [126], so that in passing from the R phase of PMN to the T of PT, the polarization does not follow the direct path but passes through an O stage. It has been proposed that the counterintuitive (from the point of view of R/T bridging) M phases of these relaxor-PT solid solutions are due to particularly soft TA phonons polarized along $\langle 110\rangle$ found in the PNR of PMN and PZN; the resulting orthorhombic strain would be responsible for the stabilization of the M${}_{C}$ phase [151].

The rotation of the polarization under the application of an electric field *E* has also been experimentally followed in single crystals. The geometry mostly used is *E* along $\left(001\right)$ of initially R parallelepipeds with $\left[100\right]$ faces; in this manner the domains with the four $\left(\pm 1,\pm 1,1\right)$ orientations are equivalent and the resulting twinning gives a pseudotetragonal symmetry. At composition well within the R phase, the rotation path of P in the initially R domains follow the straight R-T path on the application of the field, but at MPB it jumps irreversibly to the O phase and follows the M${}_{C}$ O-T path in order to approach the $\left(001\right)$ direction [8,152]. Though the phenomenology is more rich than in PZT, the fact remains that the free energy must become rather flat against changes in the direction of P near the MPB.

### 9.2. Adaptive vs. Monoclinic

As for PZT, the initial enthusiasm for the discovery of the M or O intermediate phase at the MPB [8], perfectly conforming with the explanation of high piezoelectricity from the easy rotation of the polarization [9,58], and backed by the introduction of higher order anisotropies into Landau expansion, was followed by the contrasting opinion that the apparently low symmetry phase is actually an adaptive phase. Contrasting interpretations of old and new experimental data continue to appear for all these materials, generally within the framework of genuine M phase versus adaptive phase.

For example, favoring the adaptive phase over the M is a HRTEM and convergent-beam electron diffraction study on PMN-PT, interpreted in terms of coexisting T and R nanotwinned domains [153].

On the other hand, there is additional support to the M phase also by using HRTEM and related techniques, usually used to characterize nanotwinning: Hungría *et al.* [154] found M domains in twin-free 0.39BiScO${}_{3}$-0.61PbTiO${}_{3}$ nanocrystals, with the composition tuned at the MPB. An example of how the two views of adaptive and monoclinic phases are not exclusive is a TEM study on PMN-30PT [155] where the microscale domains are found to have a regular lamellar nanostructure with spacing of 10–20 nm corresponding to nanotwinning. Yet, the orientations of these nanotwins are neither $\left\{100\right\}$ nor $\left\{110\right\}$, as expected for twins of T or R phases, and demonstrate that the symmetry of the twinned phases at the lowest scale length is M. The M nature of the nanodomains has later been unambiguously confirmed by the observation of the splittings of diffraction spots [156]. In these studies it is found that the reversible reorientation of the nanodomains under an electric field accounts for a considerable fraction of the piezoelectric effect [155,156].

### 9.3. Anelastic Spectra of PMN-PT

**Figure 16:**
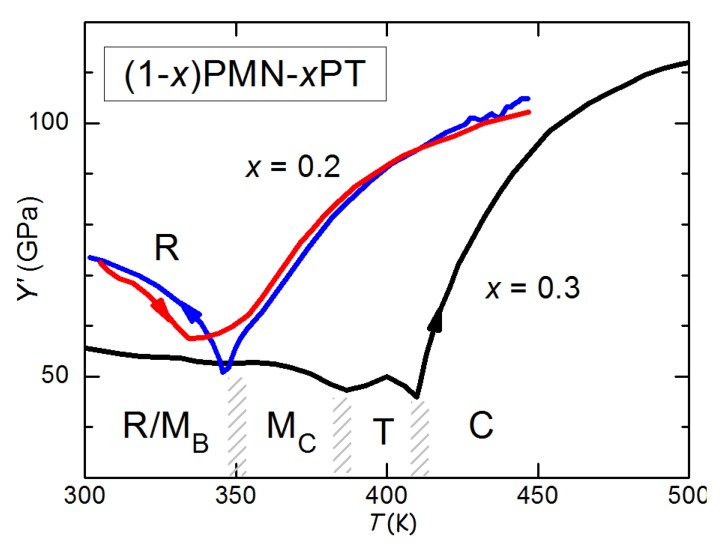
Young’s modulus of two compositions of PMN-PT measured with the DMA at 9 Hz during heating and cooling at 3 K/min (adapted from Ref. [157]). The structural phases are indicated next to the curves .

Algueró *et al.* [157] measured the anelastic spectra of PMN-20PT and PMN-30PT with the DMA at 9 Hz. According to the phase diagram of Figure 15, which presents the revision of Singh *et al.* [126] with additional M${}_{C}$ phase and R/M${}_{B}$ rather than R phase, PMN-20PT during cooling undergoes the sequence of transformations to R at $T_{C}$ and closely spaced in *T* to R/M${}_{B}$. Instead, PMN-30PT meets the sequence of transformations to T, M${}_{C}$ and M${}_{B}$. These sequences of transformations cause quite smooth and in some cases unobservable anomalies in the real part of the dielectric susceptibility, also measured on the same samples, but are better recognizable in the elastic response [157]. Figure 16 presents the $Y^{\prime }\left(T\right)$ curves of PMN-20PT and PMN-30PT measured during heating and cooling ramps at 3 K/min. Their interpretation is made in the light of the revised phase diagram [126] and of the $\chi^{\prime }\left(T\right)$ curves measured on poled PMN-PT crystals [107], where poling causes a drastic reduction of $\chi^{\prime }$ in the T phase, so rendering the M/T and T/C transitions easily recognizable. Therefore, $Y^{\prime }\left(T\right)$ of PMN-20PT measured during heating shows a minimum with superimposed a cusp, possibly related to the double C/R and R/M${}_{B}$ transition, while $Y^{\prime }\left(T\right)$ of PMN-30PT has modulations below $T_{C}$ identifiable with the transitions to T and the two M phases. The shaded areas in Figure 16 are the temperatures where $\chi^{\prime }\left(T\right)$ of poled PMN-30PT presents the signatures of these transitions [107].

The most interesting feature in the present context is the fact that while the PMN-20PT sample in the R phase partially restiffens below the initial softening from the C-PE phase, the PMN-30PT sample remains soft in the M phases. This confirms the softness of the M phase, which certainly contributes to enhancing the piezoelectric properties.

For the PMN-20PT sample both the heating and cooling ramps are shown, with a clear hysteresis in $Y\left(T\right)$: the heating curve displays a cusp superimposed to the broad minimum, that is missing in the cooling curve. This has been interpreted in terms of slow kinetics [157] that would be characteristic of relaxors, with their polar glassy dynamics [158]. Apparently, during cooling at 3 K/min there is no time for completing the transitions to the low temperature phase, while during heating, the phases that meanwhile had time to develop, transform normally producing the elastic anomalies. Something similar will be shown in Section 10 for NBT-BT.

Recently, $Y=1/s_{11}$ of a $\left(100\right)$ oriented PMN-29PT crystal in the unpoled and poled states has been measured at 0.1−10 Hz by DMA [159]. While the unpoled sample has an anelastic spectrum comparable to that of PMN-30PT [157], with even better defined steps at the FE transitions, after poling $1/Y^{\prime }$ shows a brisk decrease in the T phase, similarly to $\chi^{\prime }$.

A characteristic of the anelastic spectra of ferroelectrics with high structural and dynamical disorder, observed also in PMN-PT, is that they generally contain sharper anomalies at the various transitions with respect to the dielectric spectra.

## 10. NBT-Based Solid Solutions

### 10.1. General Properties

The solid solution (Na${}_{1/2}$Bi${}_{1/2}$)${}_{1-x}$Ba${}_{x}$TiO${}_{3}$ (NBT-BT) has an MPB at x∼0.06 between R-FE and T-FE phases [160,161] and its structural, dielectric and elastic properties are rather difficult to classify. It has considerable steric, chemical and charge disorder in the A sublattice; in addition, its end member Na${}_{1/2}$Bi${}_{1/2}$TiO${}_{3}$ (NBT) has a very small mean A ion size, that causes chemical pressure on the TiO${}_{6}$ octahedra. Therefore, during cooling from the high-temperature C-PE phase, NBT present an unusual sequence of phase transitions with tilted octahedra and accompanied by PE, AFE (or rather antiferrielectric) and finally FE cation displacements [162,163]. Already NBT presents some features classified as relaxor-like [164,165]: the dielectric susceptibility shows a rather broad peak at the R-FE/T-AFE transition with some frequency dispersion, that becomes a truly relaxor behavior on approaching the MPB composition [166,167] and beyond. Further complications come from the volatility of the Bi and Na oxides, that can introduce cation vacancies and cause deviations from the nominal stoichiometry. As a consequence, NBT-BT commonly has quite a large conductivity, that also affects the dielectric and piezoelectric properties, and requires doping, for example with Mn, in order to reduce the leakage currents [168].

Regarding the issue of adaptive R/T versus M phase, a recent study is mentioned, that enriches the phenomenology. In this study [169] NBT-7BT whiskers with diameters of 2–3 grains were prepared, in order to minimize the intergrain influence on the polarization configuration. Though according to neutron diffraction the structure was R, HRTEM revealed M twins within the R domains. This case is opposite to the usual paradigm that neutron and X-ray diffraction see an average M structure, that is locally nanotwinned R/T.

NBT can form solid solutions with other T-FE perovskites, like PT [170], which however is of comparatively little interest because of the Pb toxicity and because has lower performances than PZT, and K${}_{1/2}$Bi${}_{1/2}$TiO${}_{3}$ (KBT). Crystals of the latter system are difficult to characterize due to segregation effects from the melt, and the R/T MPB at room temperature has been reported at quite different compositions, from 17% to 80% K, depending on the sample preparation [171].

### 10.2. Elastic Properties

**Figure 17:**
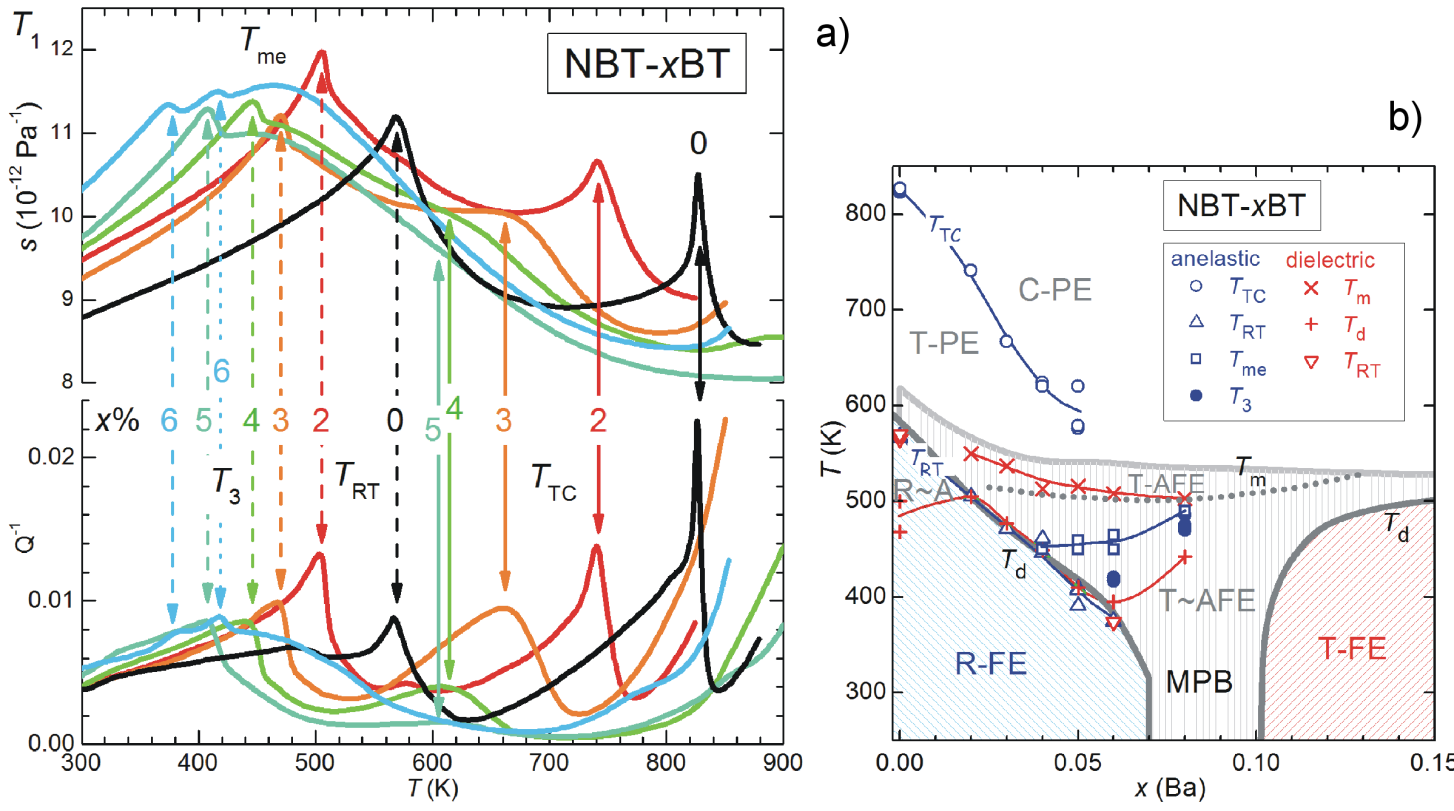
(**a**) compliance *s* (reciprocal Young’s modulus) and elastic energy loss coefficient $Q^{-1}$ of various compositions of NBT−xBT measured with the free flexural resonance at 1–5 kHz during heating. The solid arrows indicate $T_{\mathrm{TC}}$, the dashed arrows $T_{\mathrm{RT}}$ and the dotted arrow $T_{3}$. (**b**) phase diagram deduced from the anelastic and dielectric spectra [172]; the gray lines are from [160] for x<0.07 and from [173] for x>0.07.

From the point of view of the elastic properties only NBT-BT has been studied to date. Figure 17 shows a collection of anelastic spectra measured with the free flexural resonance on NBT−xBT with *x* up to 6%, close to the MPB [172]. The sharp features found in NBT are encouraging, considering that the structural transition at $T_{\mathrm{TC}}$ from cubic to T with tilted octahedra about the quaternary axis had only been observed with neutron diffraction, and the transition at $T_{\mathrm{RT}}$ from the now AFE or ferrielectric T phase (labeled T∼AFE below the temperature $T_{m}$ of the dielectric maximum in the phase diagram of Figure 17) to the R-FE phase is observed as a broad relaxor-like dielectric peak and presents a complicated phenomenology in the various diffraction experiments (for a recent survey and update see Ref. [163]).

With increasing $x\left(\text{Ba}\right)$, the anelastic spectrum soon evolves into a broad maximum of $s^{\prime }$ at $T_{\mathrm{me}}$, that evidently arises from the structural and dynamic disorder associated with the polar degrees of freedom, but still preserves one or even two sharp steps, one ($T_{\mathrm{RT}}$) identified with the structural R/T transition (octahedral tilting and cell parameters) and one labeled $T_{3}$ of still undetermined nature (such steps are visible only during heating and completely absent during cooling, compare with PMN-PT [157] in Section 9.3). It would be tempting to identify the growing maximum at $T_{\mathrm{me}}$ with the transverse instability at the MPB but the situation is more complicated. In fact, a study of this maximum by DMA at varying frequencies and *T* protocols shows that for x>0.04 the maximum at $T_{\mathrm{me}}$ can be interpreted in terms of strain glass transition [174], that prevents the prompt formation of the R phase during normal cooling (hence the absence of the steps) but has little to do with rotational instabilities of the polarization.

The disorder near the MPB of NBT-BT is so large that there is no instability in the polarization direction, because the border is between a R-FE phase at low *x* and what has been called a relaxor-AFE [173], a phase without macroscopic structural and polar domains. A regular T-FE phase is found at x>0.1, and therefore the MPB of unpoled NBT-BT is a broad region of highly disordered unpoled phase, labeled T∼AFE in the phase diagram in Figure 17. The situation changes when the material is polarized into a regular FE by application of a field of ≥3 kV/mm; in that case one has an MPB between R-FE and T-FE [175], but I am not aware of elastic studies of poled NBT-BT.

Other reports on DMA experiments have been published, where the frequency dispersion was analyzed in order to extract information on the polarization dynamics [176], and where concentrations up to $x\left(\text{Ba}\right)=0.25$ have been studied [177]. Recently, a study has been carried out of the Young’s modulus of some FE perovskites measured with the method of the impulse excitation [178]. The focus of the research is on the relaxor features of NBT-BT and NBT-KBT-BT. Again a broad minimum of the modulus is observed around ∼460 K in NBT-6BT, but rather than to the MPB it is associated with the relaxor or diffused FE transition [178].

It is possible, as for PZT in Section 8.3, to compare the kHz and lower frequency experiments on ceramics with the results of ultrasound at 1 MHz [179] and Brillouin scattering at 50 GHz [180] on crystals, where $c_{11}$ is measured. While in the case of PZT the softening at the MPB appears much more marked in the polycrystalline Young’s modulus, for NBT-BT also $c_{11}$ presents a clear minimum in the temperature region of R+T coexistence or $T_{\mathrm{me}}$. This is compatible with the disordered structure and dynamics of NBT−xBT seen as a strain glass rather than a system close to a rotational instability of the polarization.

## 11. BCTZ

**Figure 18:**
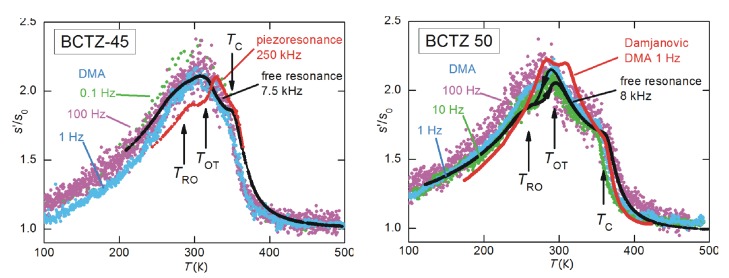
Comparison between the elastic compliances measured in different frequency ranges at two compositions of BCTZ (data from [192]. Also included are the data from DMA of Damjanovic *et al.* [187] (red curve in BCTZ-50).

The classic FE BaTiO${}_{3}$ already has many of the ingredients considered favorable to high piezoelectricity. It has the sequence of T/O/R FE phases associated with nearly isotropic free energy and hence large shear susceptibilities, such as $d_{15}$ [3]. Recently, also the M bridging phase has been found to allow the strain accommodation between the coexisting O and T domains at the T-O border [118,119,120,121], and it has also been proposed that the M phase is an adaptive nanotwinned T phase [181]. The main drawback for applications is the fact that the O-T border is a TPB to be crossed varying temperature, and therefore the high piezoelectric properties decrease fast away from $T_{\mathrm{OT}}$. Therefore, the research is active in trying to find suitable compositions based on BaTiO${}_{3}$, whose phase diagram presents some approximation of MPB between R, or better O, and T phases. A ternary system that has been studying in this respect is BaTiO${}_{3}-$ BaZrO${}_{3}-$ CaTiO${}_{3}$, where the role of the off-centering of the small Ca ions in initially stabilizing the FE-T phase is discussed in [182]. Following the study of Liu and Ren [183] on the piezoelectric properties and phase diagram of the pseudobinary system (1−x)Ba(Ti${}_{0.8}$Zr${}_{0.2}$)O${}_{3}-x$(Ba${}_{0.7}$Ca${}_{0.3}$)TiO${}_{3}$ (BCTZ−100x), much work has been done on the same and similar solid solutions. Initially it appeared that the x−T phase diagram (see Figure 18) consisted of a R-T boundary that was not as vertical as the MPB of PZT, but a reasonable approximation of it. In addition, the triple point where this $T_{\mathrm{RT}}$ line meets $T_{C}$ seems also to be a tricritical point, where the thermal hystereses of all these transitions vanish, indicating that the free energy is particularly flat for both changes of the magnitude and orientation of the polarization [183,184]. Also the elastic properties have been measured by piezoresonance on BCTZ−50 [185] and with the DMA on BCTZ−30 with 0.01Ti substituted with Sn [186], but still the maximum of the piezoelectric coefficients, which coincided with a maximum in the compliance, was associated with a R-T transition. It has to be noted that the piezoresonance experiment was conducted at widely spaced *T* intervals [185] and the DMA measurement [186] was limited to 270 K in the low *T* side.

In fact, soon DMA experiments over a broader *T* range [187] showed that there are two transitions within the FE phase of BCTZ, and the intermediate FE phase between T and R was confirmed as O by high-resolution synchrotron x-ray powder diffraction [188]. Meanwhile, experiments were aimed at establishing the role of an adaptive phase also called “domain miniaturization”. After observations by TEM of coexistence of R and T domains miniaturized down to the nanometer scale in the pseudo-MPB region [189], a similar debate as for the MPB in Pb-based materials developed. It has been proposed that the O phase is actually an adaptive phase of R and T domains [190], and strain-field loops experiments show that a major contribution to $d_{33}$ is extrinsic from DW motion [191].

A study of the role of the intermediate O phase and DW motion in enhancing the piezoelectric coefficients was carried out from the elasticity point of view, by measuring the (polycrystalline) $s_{11}$ in three frequency ranges: 1–100 Hz with a DMA, 10 kHz with flexural resonance and 250 kHz with piezoresonance [192]. The results are shown in Figure 18, where all the curves exhibit a steplike softening at $T_{C}$, followed by a peak at $T_{\mathrm{OT}}$ and a minor peak or step at $T_{\mathrm{RO}}$. In spite of the differences in techniques and frequencies the magnitudes and positions in temperature of the softenings change very little. The major changes with respect to the DMA and flexural resonance curves are in the temperatures of the softenings in the piezoresonance experiment and in that of Damjanovic *et al.* [187], but in both cases the samples were different. It is not clear if the preparation procedures may affect the properties of BCTZ, possibly through variations in the levels of cation order. At any rate, the lack of a clear and regular shift to higher *T* or reduction in amplitude of the anomalies increasing frequency over five orders of magnitude, demonstrates that the maximum of the compliance at $T_{\mathrm{OT}}$ is intrinsic.

**Figure 19:**
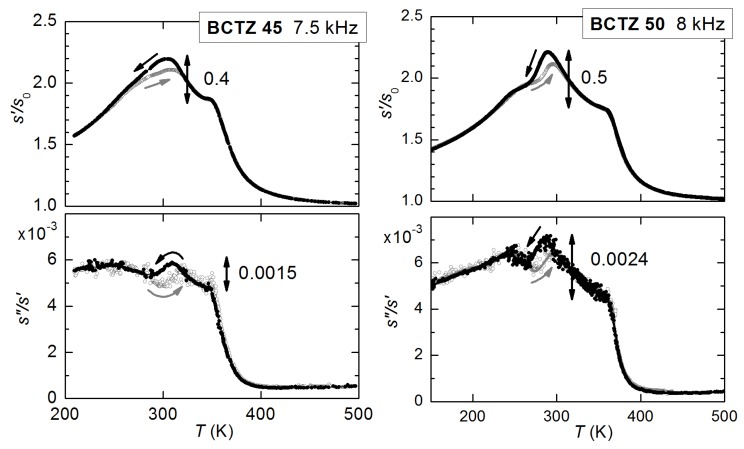
Elastic compliance and loss measured with the free resonance method in BCTZ−45 and 50 during heating and cooling (data from [192]). The double arrows indicate the amplitudes of the total contributions of the O-R anomaly to the real part and loss.

Another evidence of the intrinsic nature of the maximum in the compliance is the fact that their relative amplitudes are 200–300 times larger than the corresponding losses, as shown in Figure 19. According to Equation (14), for a relaxation process the amplitude of the step centred at ωτ=1, the real part is twice the amplitude of the peak in the imaginary part. A spectrum of relaxation times increases this ratio, since the steps are accumulated while the peaks are spread over the ωτ or *T* scale. Yet, this effect can hardly account for more than a factor of 15, obtained by assuming an unrealistically broad uniform distribution of activation energies between 0.1 and 1 eV [192].

A comprehensive analysis of the factors contributing to the large (polycrystalline) $d_{33}$ of BCTZ is described in [134], accompanied with plots of the relevant physical quantities over the x−T phase diagram based on the Landau theory. It is concluded that “trade-offs exist between reduced anisotropy energy, retention of spontaneous/remnant polarization, and elastic softening in controlling the piezoelectric activity of the BZT−xBCT system”.

**Figure 20:**
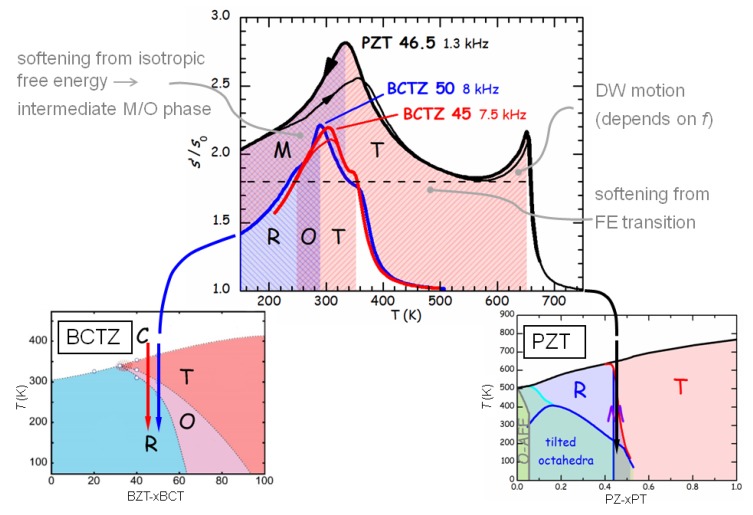
Comparison between the elastic compliances of BCTZ and PZT and paths followed in the respective phase diagrams.

It may be interesting to conclude this section with a comparison of the elastic properties and phase diagrams of PZT and BCTZ at the MPB and the O-T border (Figure 20). The dashed line is the compliance attributable to the FE-T phase in PZT; in fact, the peaked component at the FE transition is thermally activated and due to DW motion, as shown in Figure 11, while the intrinsic contribution predicted by the Landau theory, though in its simplest form, is a step (Figure 6). Assuming that this contribution remains more or less constant, the peak centered at the M/T transition can be attributed to the shear softness from the transverse instability due to the nearly isotropic free energy. Also the curves of BCTZ are normalized to their value in the PE phase, and the magnitude of the step at $T_{C}$ is very close to that in PZT, but the peak at the transition to the intermediate O phase is less than half, and also its extension in temperature is more limited than in PZT. The fact that the peak at the intermediate transition is limited with respect to PZT is an indication that the transverse instability of the polarization, which is coupled to the shear compliance, is less pronounced, and this is also in accordance with the fact that the intermediate phase is O rather than M. While PZT is closer to having MPB where the structure is tuned close to a transverse instability, BCTZ undergoes the same sequence of phase transitions as BaTiO${}_{3}$, only better tuned to room temperature.

## 12. KNN-Based Solid Solutions

Also the K${}_{1-x}$Na${}_{x}$NbO${}_{3}$ (KNN) solid solution presents several MPBs, particularly between M Pm and O Amm2 phases at x=0.5, a composition often in connection with piezoelectric applications, where by KNN it is usually meant K${}_{0.5}$Na${}_{0.5}$NbO${}_{3}$. This boundary seems also really vertical in the x−T phase diagram, but its influence on the piezoelectric properties is scarse, because it separates phases differing in the octahedral tilt system, but with little change of the polarization [115].

By alloying with other components, it is possible to obtain also boundaries between FE phases differing more in the orientations of P. The phase diagram of KNN−xLiNbO${}_{3}$ [193] has the usual sequence of R/O/T/C phases as BaTiO${}_{3}$ with rising temperature, when $x\left(\text{Li}\right)<0.07$. Alloying with LiSbO${}_{3}$ lowers $T_{\mathrm{OT}}$, which we have seen is accompanied by considerable lowering of the anisotropy, closer to room temperature. A comparative study of the piezoelectric, dielectric and elastic responses has been done for KNN−5.2LiSbO${}_{3}$ [194], and it was found that the peak in *d* was accompanied by peaks in *ε*, *s* and remnant polarization, so that all these properties contribute to enhancing piezoelectricity, but the decay away from $T_{\mathrm{OT}}$ was more marked than for the MPB of PZT.

Recently, a study including the elastic properties measured with DMA has been conducted in the multinary solid solution [(K${}_{0.5}$Na${}_{0.5}$)${}_{0.95}$Li${}_{0.05}$Nb${}_{1-x}$Sb${}_{x}$O${}_{3}]-$ 4BaZrO${}_{3}$ [195]. Its $x\left(\text{Sb}\right)-T$ phase diagram has again the sequence of R/O/T/C phases with rising temperature, and again the crossing of the O/T boundary is accompanied by peaks in the susceptibilities.

**Figure 21:**
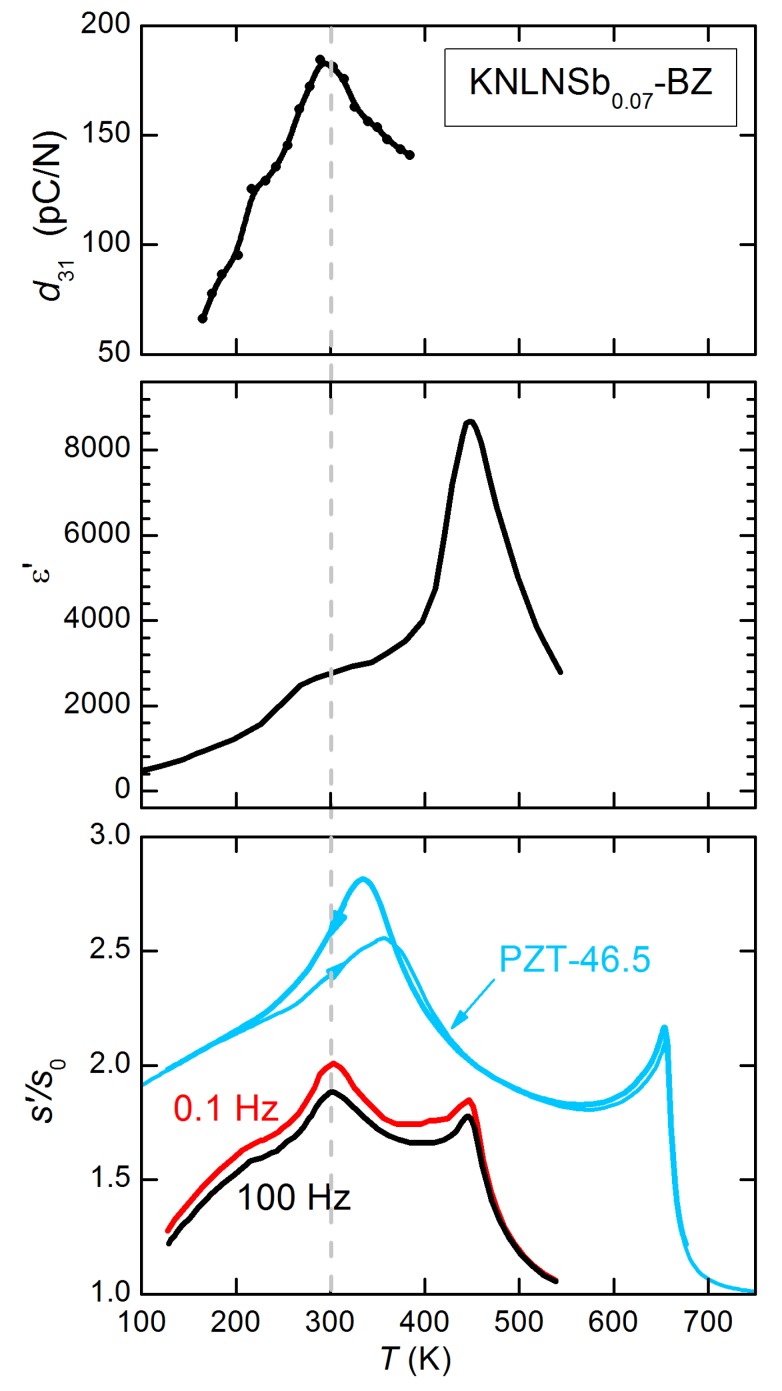
Elastic compliance $s^{\prime }$, dielectric susceptibility $\varepsilon^{\prime }$ and piezoelectric coefficient $d_{31}$ of KNLNSb${}_{0.07}-$BTZ (data extracted from [195]). The compliance is normalized to its value in the PE phase and compared with that of PZT-46.5 from Figure 12 and Figure 20.

A comparison of the *d*, *ε* and *s* curves versus *T*, extracted from [195], is reproduced in Figure 21, that also includes a comparison with the compliance of PZT−46.5, whose $T_{\mathrm{MPB}}$ is close to $T_{\mathrm{OT}}$. The compliance *s* is the reciprocal Young’s modulus measured with the DMA at frequencies between 0.1 and 100 Hz and is normalized to its value in the PE phase. The comparison with PZT is similar to the BCTZ case in Figure 20: the softening at $T_{C}$ is close to that of PZT, but the amplitude of the peaked softening at $T_{\mathrm{OR}}$ is less than 1/3 than in PZT and in a more limited temperature range. From the dispersion over three decades of frequency in the $s^{\prime }\left(T\right)$ curves it is clear that the DW motion produces some softening below $T_{C}$ but definitely not the peak at $T_{\mathrm{OT}}$. In fact, the difference between the low- and high-frequency curves is constant, with no change in position, shape or intensity of the peak at $T_{\mathrm{OT}}$. The comparison with the $\varepsilon^{\prime }$ and *d* curves shows that the elastic compliance is the main source of enhancement of $d_{31}$.

## 13. Conclusions

The study of the boundaries between FE phases is fundamental in understanding the mechanisms and improving the materials with high piezoelectric response. In fact, the proximity to such phase boundaries provides the conditions for high piezoelectric coefficients: high susceptibilities, usually with respect to changes of the direction of the polarization and to shear strain, but also large spontaneous polarization. In addition, if the boundary is close to vertical in the composition-temperature phase diagram, it is called morphotropic and the properties depend more on changes of composition than of temperature, and this implies a better temperature stability. These conditions are met mainly in solid solutions of PbTiO${}_{3}$ with AFE PbZrO${}_{3}$ and relaxor Pb(B${}^{\prime },$B${}^{\prime \prime }$)O${}_{3}$ compounds, but also of Na${}_{1/2}$Bi${}_{1/2}$TiO${}_{3}$ and of NaNbO${}_{3}$ with other Pb-free perovskites. These classes of materials present similarities, like M or O phase that are intermediate between the two (or more) alloyed FE phases, and fine and complex domain structures, but also present peculiarities. A major issue is to separate the contribution to the enhancement of the piezoelectric effect from the intrinsic properties of the intermediate phases and from the extrinsic motions of domain walls.

The measurement of the complex dynamic compliance (or modulus) is not a routine characterization of ferroelectric materials, but is complementary to and as important as the dielectric characterization for various reasons: (i) the elastic compliance contributes to the piezoelectric effects in the same manner as the dielectric susceptibility, or in simple words, a softer material is a better piezoelectric; (ii) the combined knowledge of dielectric and elastic responses provides information on the nature of the structural phases and their transitions, that are otherwise difficult to obtain in complex materials with domain nanostructures; (iii) being insensitive to the polar dynamics, the elastic response is not dominated by the Curie-Weiss peak of the main FE transition as the dielectric susceptibility is, and hence is more sensitive to all the other transitions, notably that at the MPB (see e.g., PZT, Figure 12); (iv) being insensitive to free charges, the elastic compliance provides useful information also in highly defective materials, with apparently poor but potentially high piezoelectric coefficients after proper optimization.

The most complete elastic and anelastic studies of ferroelectrics near their MPB are on PZT, where it is shown that the major softening peaked at the MPB demonstrates that there is an intermediate M phase, rather than a purely nanotwinned adaptive phase with local R or T symmetry. In addition, other new features of the phase diagram of PZT have been found, thanks to the sensitivity of the elastic response to octahedral tilting.

Important results regarding the mechanisms behind a large piezoelectric response have been obtained also in BCTZ, that, though not having a close to vertical MPB in the composition−T phase diagram, shares much physics and phenomenology with systems with MPB. In other classes of materials like relaxor-PT, NBT-BT and KNN-based, there are no systematic studies of the elastic properties, but some studies with limited scope, yet with interesting and promising results.

It is suggested that, for the study and improvement of the piezoelectric properties, it would be generally very useful to complement the traditional dielectric, ferroelectric and structural methods with characterization of elastic and anelastic properties.

## Abbreviations and Conventions

The Voigt notation for the stress/strain and sometimes for the double polarization variables is used starting from Section 5.1; it transforms the double tensor index ij into a single index *i*: 11→1, 22→2, 33→3, 23→4, 13→5, 12→6.

## Phases

AFE	antiferroelectric
C	cubic
FE	ferroelectric
M	monoclinic
O	orthorhombic
MPB	morphotropic phase boundary
PE	paraelectric
R	rhombohedral
T	tetragonal
TPB	thermotropic phase boundary
$T_{C}$	Curie temperature
$T_{\mathrm{MPB}}$	temperature of the MPB, usually $=T_{\mathrm{MT}}$ or $T_{\mathrm{OT}}$
$T_{T}$	temperature below which the octahedra are tilted
$T_{\mathrm{XY}}$	temperature of the transition between the low-*T* phase X and the high-*T* phase Y

## Physical Quantities

$c_{ijkl}$, $c_{ij}$ (Voigt)	elastic stiffness constant
$d_{ijk}$, $d_{ij}$ (Voigt)	piezoelectric constant
$e_{ij}$, $e_{i}$ (Voigt)	strain
$s_{ijkl}$, $s_{ij}$ (Voigt)	elastic compliance
$\sigma_{ij}$, $\sigma_{i}$ (Voigt)	stress
$\chi_{ij}$	dielectric susceptibility
*F*	Helmholtz free energy
*G*	shear modulus
G=F−σe	Gibbs free energy
P, $P_{i}$	(spontaneous) polarization
$Q^{-1}=s^{\prime \prime }/s^{\prime }$
$=c^{\prime \prime }/c^{\prime }$	elastic energy loss coefficient
$Q_{ijkl}$, $Q_{ij}$ (Voigt)	electrostrictive coefficient
*Y*	Young’s modulus

## Materials

ABC−100xDEF	(1−x)ABC−xDEF
BCTZ−100x	(1−x)Ba(Ti${}_{0.8}$Zr${}_{0.2}$)O${}_{3}-x$(Ba${}_{0.7}$Ca${}_{0.3}$)TiO${}_{3}$
PMN	Pb(Mn${}_{1/3}$Nb${}_{2/3}$)O${}_{3}$
PSN	Pb(Sc${}_{1/2}$Nb${}_{1/2}$)O${}_{3}$
PZN	Pb(Zn${}_{1/3}$Nb${}_{2/3}$)O${}_{3}$
PT	PbTiO${}_{3}$
PZT-100x	PbZr${}_{1-x}$Ti${}_{x}$O${}_{3}$

## Techniques

DMA	Dynamic Mechanical Analyzer
HRTEM	High Resolution Transmission Electron Microscopy

## Other Abbreviations

DW	domain wall
PNR	Polar Nano Regions
XRD	X-Ray Diffraction
